# The CRISPR-associated adenosine deaminase Cad1 converts ATP to ITP to provide antiviral immunity

**DOI:** 10.1016/j.cell.2024.10.002

**Published:** 2024-10-28

**Authors:** Christian F. Baca, Puja Majumder, James H. Hickling, Linzhi Ye, Marianna Teplova, Sean F. Brady, Dinshaw J. Patel, Luciano A. Marraffini

**Affiliations:** 1 Laboratory of Bacteriology, The Rockefeller University, New York, NY 10065, USA; 2 Tri-Institutional PhD Program in Chemical Biology, Weill Cornell Medical College, Rockefeller University and Memorial Sloan Kettering Cancer Center, New York, NY 10065, USA; 3 Structural Biology Program, Memorial Sloan-Kettering Cancer Center, New York, NY 10065, USA; 4 Laboratory of Genetically Encoded Small Molecules, The Rockefeller University, New York, NY 10065, USA; 5 Howard Hughes Medical Institute, The Rockefeller University, New York, NY 10065, USA; 6 These authors contributed equally; 7 Lead contact

## Abstract

Type III CRISPR systems provide immunity against genetic invaders through the production of cyclic oligoadenylate (cA_n_) molecules that activate effector proteins that contain CRISPR-associated Rossman fold (CARF) domains. Here, we characterized the function and structure of an effector in which the CARF domain is fused to an adenosine deaminase domain, CRISPR-associated adenosine deaminase 1 (Cad1). We show that upon binding of cA_4_ or cA_6_ to its CARF domain, Cad1 converts ATP to ITP, both *in vivo* and *in vitro*. Cryoelectron microscopy (cryo-EM) structural studies on full-length Cad1 reveal an hexameric assembly composed of a trimer of dimers, with bound ATP at inter-domain sites required for activity and ATP/ITP within deaminase active sites. Upon synthesis of cA_n_ during phage infection, Cad1 activation leads to a growth arrest of the host that prevents viral propagation. Our findings reveal that CRISPR-Cas systems employ a wide range of molecular mechanisms beyond nucleic acid degradation to provide adaptive immunity in prokaryotes.

## In brief

Cad1 is an hexameric type III CRISPR-CARF effector harboring an adenosine deaminase domain that is activated by cyclic oligonucleotides produced upon phage recognition. Cad1 activation results in ATP deamination, accumulation of ITP, and a growth arrest of the infected host that prevents viral spread.

## Graphical Abstract

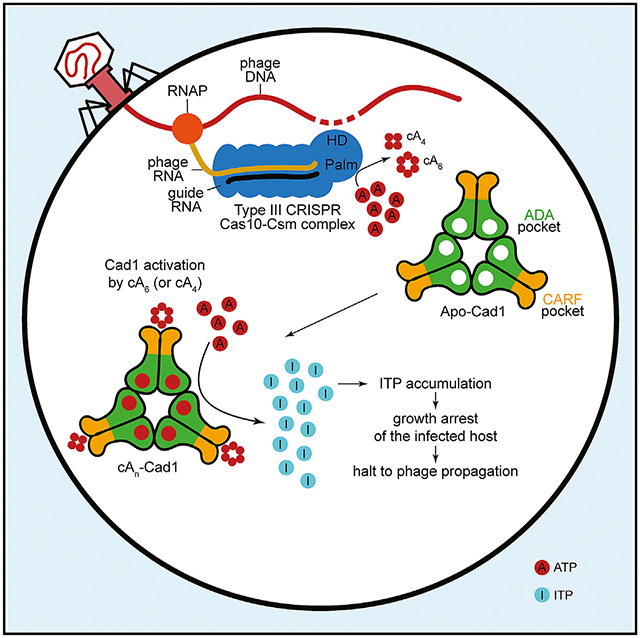

## INTRODUCTION

Clustered regularly interspaced short palindromic repeat (CRISPR) loci provide adaptive immunity to bacteria and archaea against phages^1^ and plasmids.^2^ In these loci, DNA repeats are separated by short (~30 bp) “spacer” sequences acquired from foreign genetic elements during infection.^1^ CRISPR arrays are transcribed and processed into CRISPR RNAs (crRNAs) that are loaded onto an effector complex^3–5^ and used as guides to recognize invading complementary DNA or RNA sequences to start the CRISPR immune response.^6–8^ Type III CRISPR-Cas systems encode an effector complex in which Cas10 is the main subunit that recognizes RNA targets^7^ to trigger two activities: single-stranded DNA (ssDNA) degradation^9^ and synthesis of second messengers known as cyclic oligoadenylates (cOAs).^10,11^ The first activity is catalyzed by the HD domain of Cas10^9^ and is sufficient to provide anti-phage immunity when the target viral transcript is expressed early, but not late, in the lytic cycle.^12,13^ By contrast, cOA production is carried out by the Palm domain of Cas10^10,11^ to create a 3′–5′ cOA molecule using ATP as substrate (with cA_4_ and cA_6_ being the most abundant species), and it is essential for defense when the crRNA is complementary to a late-expressed phage RNA.^12–14^ In this case, immunity is carried out through the activation of CRISPR-associated Rossman fold (CARF) effectors by the cOA second messenger. In addition to the CARF domain that binds the cOA ligand, these effectors possess a second domain that causes cell toxicity, preventing the host from supporting the phage’s lytic cycle.^12,13,15–17^

## RESULTS

### Cad1 activation leads to growth arrest and high levels of cellular ITP

**Figure 1.**
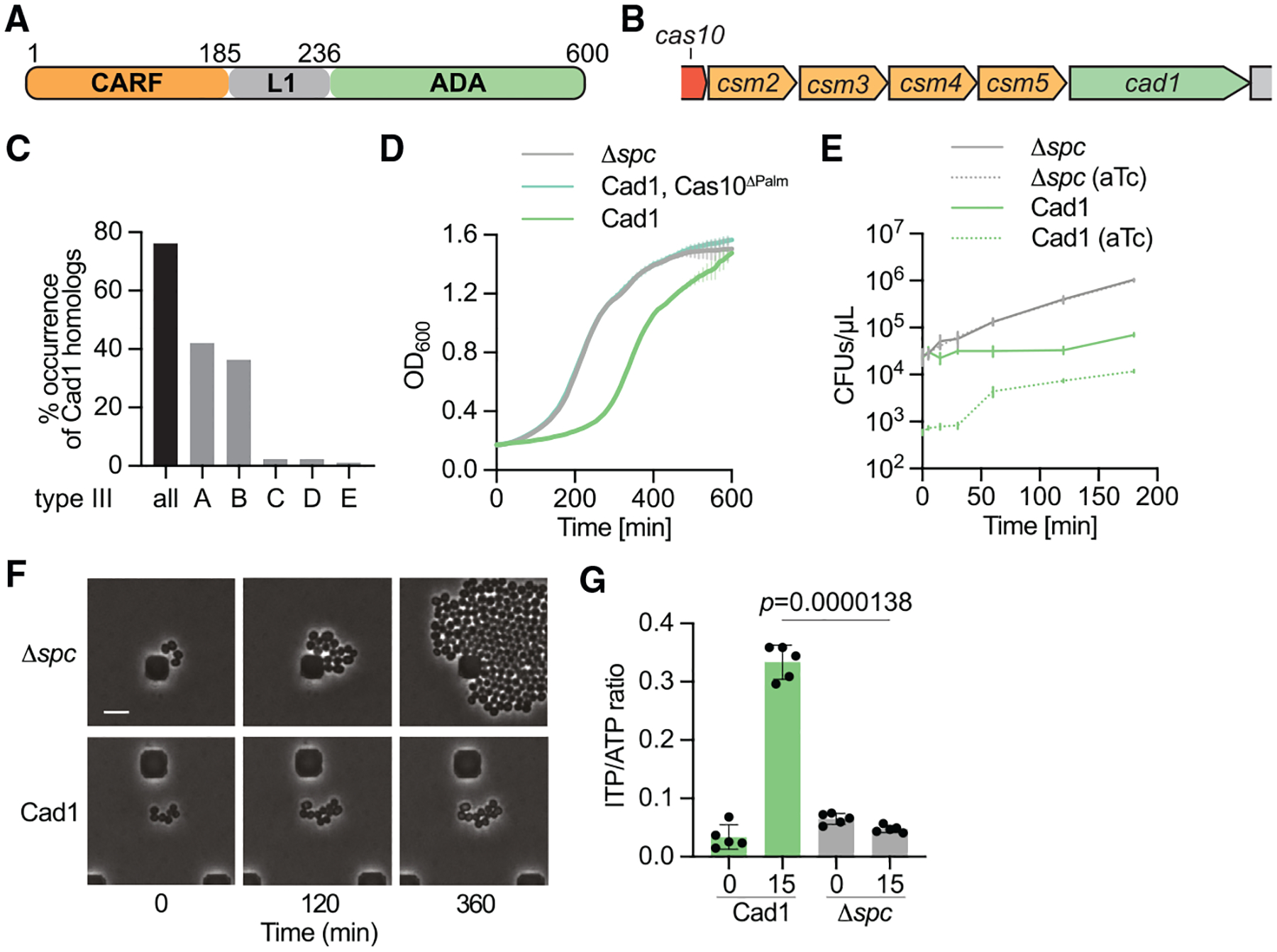
Cad1 activation leads to growth arrest and high levels of cellular ITP (A) Domain architecture of *Bacteroidales* Cad1. The protein contains a N-terminal CARF domain followed by a linker (L1) and a C-terminal adenosine deaminase (ADA) domain. Residue numbers are labeled. (B) Cad1 used in this study was identified within a type III CRISPR-*cas* locus in a contig from an unknown *Bacteroidales* bacterium. (C) Association of Cad1 homologs with different subtypes of type III CRISPR-Cas systems (88 non-redundant homologs used for quantification). (D) Growth of staphylococci carrying pTarget and different pCRISPR variants, measured as OD_600_ after the addition of aTc. Mean of three biological triplicates, ±SEM, is reported. (E) Enumeration of colony-forming units (CFUs) from staphylococcal cultures carrying different pCRISPR variants after the addition of aTc. At the indicated times after induction, aliquots were removed and plated on solid medium with or without aTc to count the remaining viable cells. Mean of three biological replicates, ±SEM, is reported. (F) Time course microscopy of *S. aureus* cells harboring pTarget and pCRISPR(Δspc) or pCRISPR(Cad1) at different times after addition of aTc, experiment repeated for two biological replicates. Scale bar, 4 μM. (G) Quantification of ITP/ATP ratios from bacterial lysates. Extracts from staphylococci harboring pTarget and pCRISPR(Δspc) or pCRISPR(Cad1) were either collected before (0 min) or after (15 min) incubation with aTc and analyzed via LC-MS. Mean of five biological replicates ±SEM, is reported. *p* values, obtained with a two-sided t test with Welch’s correction, are shown. See also Figures S1 and S2.

In order to identify novel CARF effectors, we used Foldseek^18^ to search for proteins with structural homology to CARF domain of the Cam1 effector.^12^ This method rendered a 600-residue protein containing an N-terminal CARF domain followed by a linker region and a C-terminal adenosine deaminase (ADA) domain (Figure 1A), with accession number HAJ98955.1 from an unknown *Bacteriodales* bacterium (hereafter named CRISPR-associated deaminase, CRISPR-associated adenosine deaminase 1 [Cad1]), for which homologs were already identified in a previous bioinformatic screen.^19,20^ *cad1* is located immediately downstream the *cas10-csm* genes of a type III-A CRISPR locus (Figure 1B). Further analysis of *cad1* homologs showed that over 70% associate in genomes containing type III systems (mostly belonging to the A and B subtypes, Figure 1C) and exist within many unique phyla of bacteria (Figure S1A). These data suggest that Cad1 is a widespread effector in type III CRISPR-Cas systems.

To investigate the activity and function of Cad1 *in vivo*, we cloned the *Staphylococcus epidermidis* RP62 type III-A CRISPR-*cas* locus into the staphylococcal vector pC194^21^ and replaced the *csm6* CARF effector of this locus for *cad1*, generating pCRISPR (Figure S1B). Importantly, the bacteriodales type III-A *csm* genes share 35%–51% sequence homology at the protein level with the staphylococcal *csm* genes (Figure S1C). Given that most CARF effectors characterized to date cause cell toxicity when activated,^12,13,15–17,22^ we tested whether Cad1 induction by cOAs generates a growth arrest. To do this, we introduced pCRISPR along with a second plasmid expressing a target transcript under the control of a tetracycline-inducible promoter (pTarget)^22^ into *Staphylococcus aureus* RN4220.^23^ Addition of anhydrotetracycline (aTc) produces a target transcript, which in turn leads to cOA synthesis upon its recognition by the crRNA guide within the Cas10 complex. In order to prevent degradation of pTarget by the ssDNase activity of Cas10,^22^ we mutated the catalytic residues of the HD domain^9^ (*cas10*^HD^, harboring H14A and D15A substitutions, Figure S1B). A pCRISPR plasmid lacking a targeting spacer (Δ*spc*) or carrying inactivating mutations within Cas10’s palm domain^10,11^ (*cas10*^palm^, D586A, and D587A) were used as negative controls (Figure S1B). We then measured the optical density at 600 nm (OD_600_) of different cultures upon addition of aTc and detected a notable growth delay that was not observed in cells carrying Cas10^palm^ nor Δ*spc* plasmids (Figure 1D). Eventually, cultures with active Cad1 increase in OD_600_, most likely a result of the propagation of “escaper” cells carrying non-functional pCRISPR plasmids that was previously reported for similar experiments with other CARF effectors.^12,13,22^ We also tested a Cad1 homolog from *Desulfomicrobiaceae* bacterium, which has a similar domain architecture (Figure S2A) but relatively low sequence similarity with *Bacteriodales* Cad1 (Figure S2B). Using the toxicity assay, we found that this homolog causes a similar growth arrest to staphylococci that express a target transcript (Figure S2C).

A delay in the increase of OD_600_ values could be due to either the arrest or the death of individual cells within the culture upon Cad1 activation. To distinguish between these possibilities, we quantified viable staphylococci after addition of aTc, plating aliquots taken from liquid cultures at different times on solid media lacking the inducer (Figure 1E). Absence of aTc would allow the formation of colonies by cells that were arrested in the liquid culture, but not by those that died after Cad1 activation. We observed a stable number of colony-forming units (CFUs), demonstrating the presence of a population of viable cells that cannot grow but do not die upon induction of Cad1 activity. By contrast, due to lack of cOA production, Δ*spc* cultures produced increasing colony counts. We also enumerated CFUs resistant to aTc induction (Figure 1E) and found that approximately 10% of the colonies that are formed after de-activation of Cad1 originate from escaper cells (see above). Finally, using live microscopy, we observed that while cells that cannot activate Cad1 (Δ*spc*) grow continuously (Figure 1F), cells producing cOAs proliferate at a very low rate (Figure 1F).

ADAs convert adenosine molecules to inosine (Figure S2D). Therefore, we tested the prediction that, due to the presence of the ADA domain, activation of Cad1 by cOAs results in changes in the adenosine and/or inosine cell content during the type III-A response. To do this, we collected staphylococcal lysates either before or 15 min after Cad1 activation by addition of aTc, extracted small molecules, and performed a liquid chromatography-mass spectrometry (LC-MS) analysis to quantify adenosine and inosine-containing molecules (Figure 1G). We observed an approximately 10-fold increase in the ITP/ATP ratio after addition of aTc, which was not detected in staphylococci that do not activate cOA production due to the lack of a targeting spacer (Δ*spc*). Interestingly, the increase is a consequence of the presence of large amounts of ITP (Figure S2E) but is not accompanied by a decrease in ATP levels (Figure S2F). By contrast, the levels of dITP and UTP in the induced lysates did not increase (Figures S2G and S2H), suggesting that Cad1 does not deaminate dATP nor CTP nucleotides. Altogether, these data demonstrate that cOA production during the type III-A CRISPR-Cas response activates Cad1, leading to high cellular levels of ITP and a growth arrest of the host that is reversible and does not result in cell lysis.

### cA_4_ and cA_6_ bind to the CARF domain of Cad1

**Figure 2.**
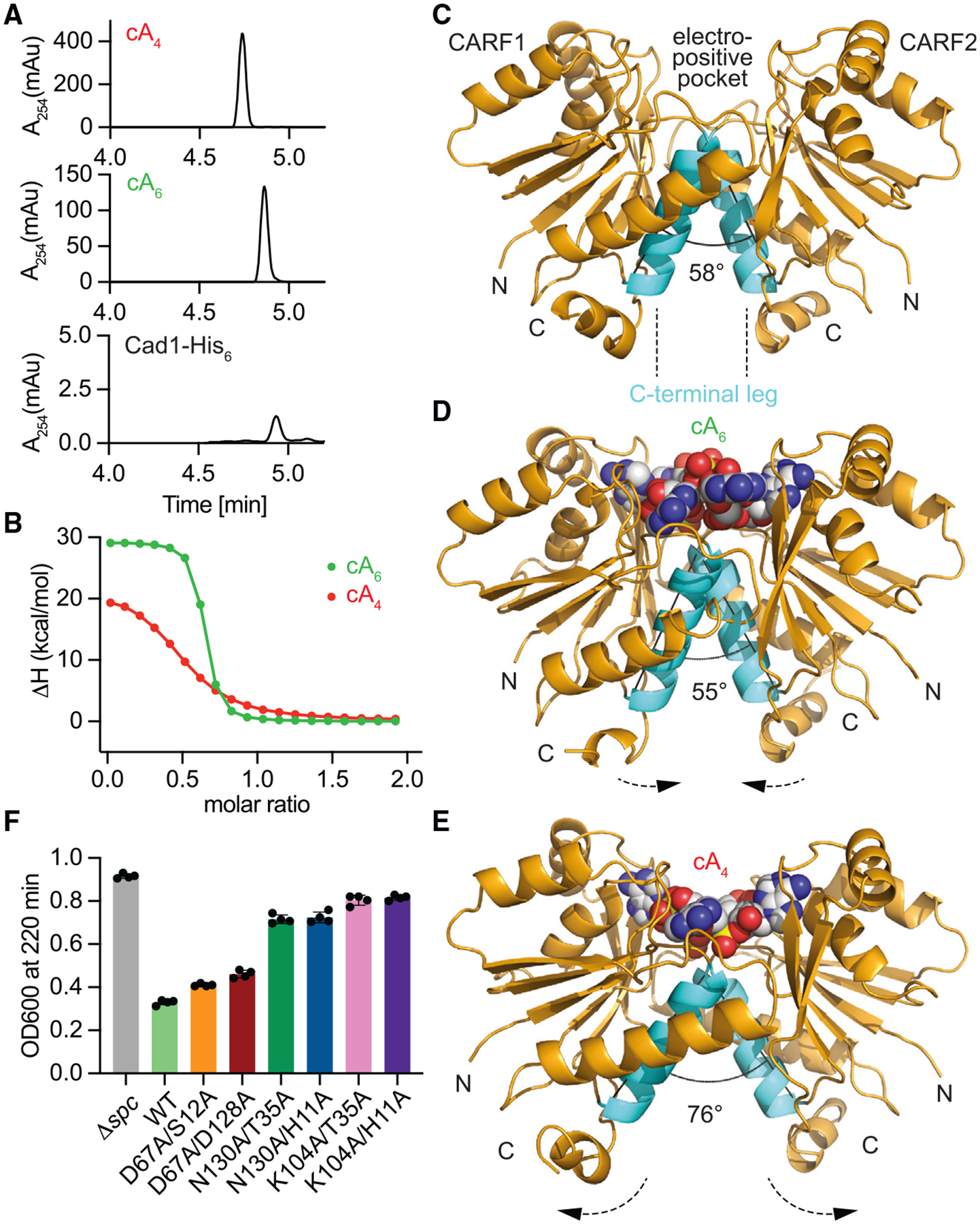
cA_4_ and cA_6_ bind to the CARF domain of Cad1 (A) HPLC analysis of cOAs associated with purified Cad1-His_6_. cA_4_ and cA_6_ standards were used as controls. (B) ITC binding curve of Cad1-CARF-His_6_ to cA_4_ and cA_6_ representing the FitX and FitY values estimated by MicroCal PEAQ-ITC analysis software (Malvern). *K*_d_ values are ~700 and ~30 nM, respectively. (C) Crystal structure of dimeric apo-Cad1-CARF-His_6_. The predicted ligand binding pocket is shown, and the C-terminal legs from each monomer are colored in cyan. The angle between the C-terminal legs is 58°. (D) cA_6_-bound structure of Cad1-CARF-His_6_ showing the ligand at the dimeric interface of the CARF domains, with the angle between the C-terminal legs becoming 55°. (E) cA_4_-bound structure of Cad1-CARF-His_6_ showing an increase in the spread of the C-terminal legs to 76°. (F) Growth of staphylococci carrying pTarget and pCRISPR(Cad1) harboring alanine substitutions of Cad1 residues involved in cOA binding, measured as the OD_600_ value after 220 min of addition of aTc. Mean of four biological replicates, ±SEM, is reported. See also Figure S3.

To determine which second messenger activates deamination *in vivo*, we added of a C-terminal hexa-histidyl tag to Cad1 (Cad1-His_6_), which did not alter its toxic properties after addition of aTc (Figure S3A). We then purified active Cad1-His_6_ following induction of target transcription (Figures S3B and S3C). The protein preparation was heated at 85°C to denature Cad1-His_6_ and release its ligands. The resulting solution was filtered through a ten kDa filter to remove denatured proteins, and the filtrate was subjected to high-performance liquid chromatography (HPLC) separation using cA_4_ and cA_6_ standards. We obtained a small but clear peak with the same retention time as cA_6_ (Figure 2A), a result that indicates that the hexameric form of the cyclic adenylate is the main ligand of Cad1 in staphylococci. To further test this finding, we purified a hexa-histidyl-tagged CARF domain (residues 1–185, Cad1-CARF-His_6_) and tested the binding of different cOAs using isothermal titration calorimetry (ITC) (Figure 2B). We found that Cad1-CARF-His_6_ binds to both cA_6_ and cA_4_ with a *k*_*D*_ of 30 and 700 nM, respectively. Next, we used X-ray crystallography to elucidate the molecular details of cOA interactions with Cad1’s CARF domain. We first solved the crystal structure of apo-Cad1-CARF-His_6_ at 3.6 Å resolution. The structure showed an asymmetric unit composed of a tetramer formed by crystal packing between two Cad1-CARF-His_6_ dimers (Figures 2C and S2D) in which monomers are arranged in a pseudo-2-fold symmetry (Figure S2E). Consistent with these results, SECMALS analysis of Cad1-CARF-His_6_ showed the formation a stable dimer in solution (Figure S2F). Analysis of the electrostatic potential revealed the presence of a positively charged pocket (Figure S2G) that harbors both K104 residues on top of the C-terminal legs (residues 104–117) on each of the CARF monomers at the dimeric interface (Figures S3E and S3G).

We also co-crystallized cA_6_ and cA_4_ with Cad1-CARF-His_6_ and solved the crystal structures at 1.8 and 2.4 Å resolution, respectively (Figures 2D and 2E). Although both cOA ligands were fully accommodated in the electropositive pocket of apo Cad1-CARF-His_6_ (Figures 2D, 2E, and S2G), they affected differently the angle between the two C-terminal legs of the CARF monomers. While cA_6_ binding produced a minor change (from 58° in the apo structure to 55°), cA_4_ binding opened this angle to 76°, most likely a consequence of the deep burial of cA_4_ within the pocket (Figures 2C–2E). The Watson-Crick edges of several adenosines of bound cA_6_ and cA_4_ form intermolecular hydrogen bonds group of charged residues within the pocket, including K104, that account for the specificity of cOA recognition (Figures S2H and S2I). To test the importance of these residues for Cad1 activation, we introduced alanine substitutions of K104, D67, H11, S12, N130, T35, and D128 and induced a growth arrest of cultures harboring different combinations of these mutations. While N130A/T35A, N130A/H11A, K104A/T35A, and K104A/H11A double mutants abrogated Cad1 activation, D67A/S12A and D67A/D128A mutations did not alter the growth arrest produced by Cad1 (Figure 2F). In addition, none of the single alanine mutants alter Cad1’s ability to slow the growth of staphylococci (Figure S2J). Given that the *S. epidermidis* Cas10 complex is able to produce both ligands at the same time,^11–13^ it is possible that only substitutions in residues important for the binding of both cA_6_ and cA_4_ prevent Cad1’s activation *in vivo*, leading to this seemingly high tolerance to mutations in the binding pocket.

### Cad1 adopts a trimer of dimers topology

**Figure 3.**
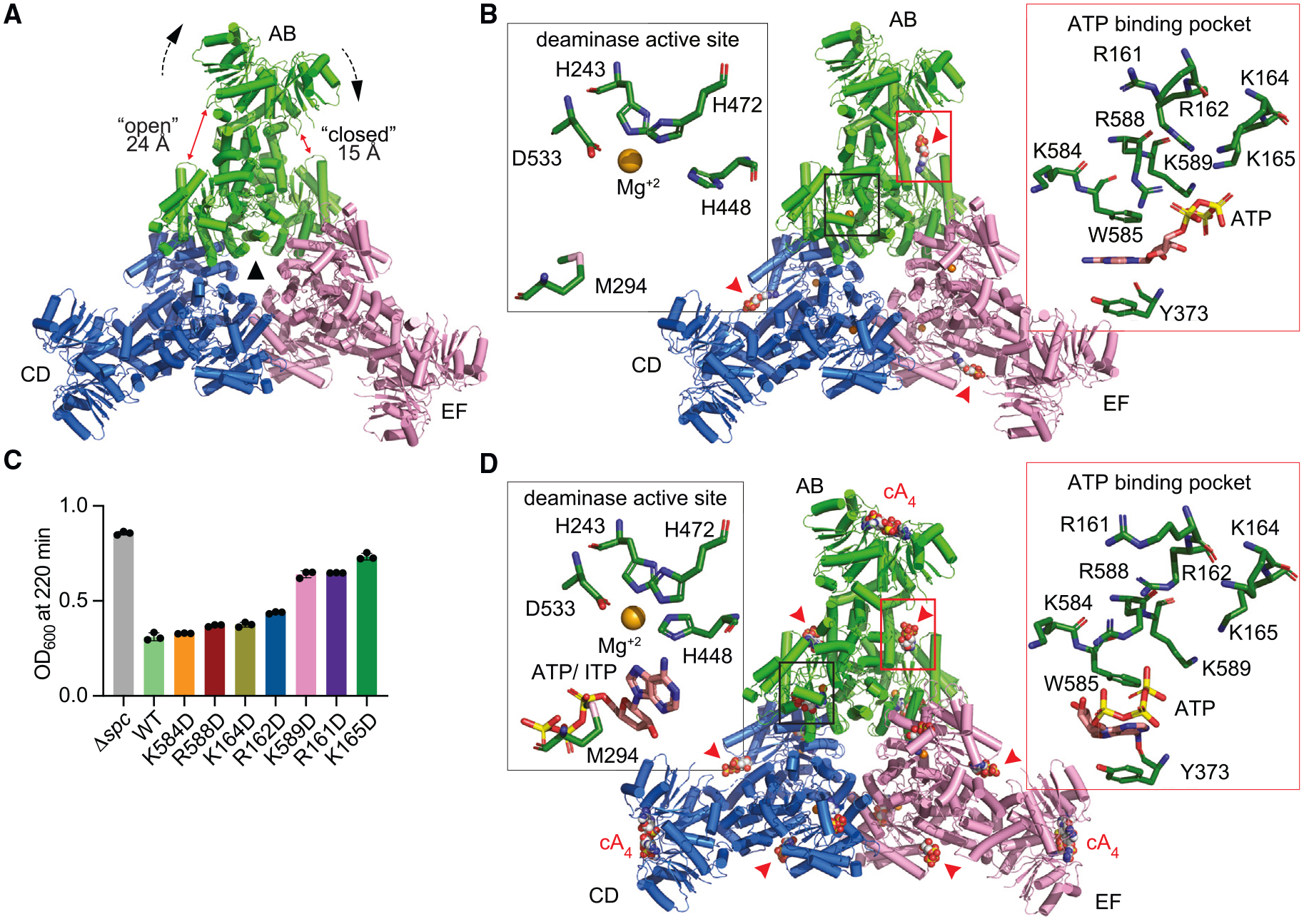
Cad1 adopts a trimer of dimers topology (A) Cryo-EM structure of hexameric apo-Cad1 illustrating the arrangement of AB, CD, and EF Cad1 dimers (in green, blue, and pink, respectively) aligned in a 3-fold symmetric arrangement (black center triangle). A pocket is formed on the interface of the CARF and ADA domains, and the distance between these domains is 15 Å (closed) on one side of the Cad1 dimer and increases to 24 Å (open) on the opposite side. The pocket is generated by the tilting of the CARF head domain (black dotted arrows). (B) Cryo-EM structure of ATP-bound hexameric Cad1 (conformation 1) displaying AB, CD, and EF dimers in green, blue, and pink, respectively. Insets highlight the residues at one of the inter-domain ATP binding sites (red border, with red arrowheads pointing at the ATP) and one of the empty deaminase pockets (black border). The metal is modeled as Mg^+2^. (C) Growth of staphylococci carrying pTarget and pCRISPR(Cad1) harboring the amino acid substitutions of the residues lining the inter-domain ATP binding pocket, measured as the OD_600_ value after 220 min of addition of aTc. Mean of three biological triplicates, ±SEM, is reported. (D) Cryo-EM structure of hexameric Cad1 protein in presence of ATP and cA_4_, displaying AB, CD, and EF dimers in green, blue, and pink, respectively. cA_4_, ATP, and ATP/ITP molecules are shown in space-filling representation. Insets highlight the residues at one of the inter-domain ATP binding sites (red border, with red arrowheads pointing at the ATP) and one of the deaminase pockets (black border). The metal is modeled as Mg^+2^. See also Figures S4, S5, S9, and S10.

Next, we characterized the structural and biochemical properties of Cad1-His_6_. First, we used SECMALS to analyze the oligomeric state of the protein and found that the majority of Cad1 forms a hexameric (403 kDa ± 0.451%) species in solution, with a small amount of the dimeric (139 kDa ± 1.1%) species and of a higher molecular weight (5–31 MDa) megadalton fraction (Figure S4A, peaks “H,” “D,” and “M,” respectively). To understand the organization of the protein at the atomic level, we solved the cryoelectron microscopy (cryo-EM) structure of hexameric apo-Cad1 at 3.6 Å resolution (Figures 3A and S4B). Consistent with the SECMALS data, the structure displayed a trimeric arrangement of Cad1 dimers forming a homohexamer. The dimeric deaminase domains form the core of the hexamer by arranging themselves with 3-fold symmetry and are linked to the CARF head domains through a 51-amino-acid linker (L1, inset, Figure S4B). Further, we observed that the dimeric CARF head groups are tilted to one side with respect to a vertical axis from the center of 3-fold symmetry (black dotted arrows, Figure 3A). The tilting of the CARF domain around the L1 linker brings it close to the deaminase domain on one side and distant from the deaminase domain at the opposite side (labeled as “closed” and “open,” red arrows, Figure 3A). Given that ATP is the putative substrate of Cad1, we obtained cryo-EM structures in the presence of this molecule and found two different conformations with either symmetric (3.4 Å resolution, Figure 3B) or asymmetric (3.6 Å resolution, Figure S4C) positioning of three ATPs. In both cases, one ATP molecule was bound per Cad1 dimer, unexpectedly not in the deaminase domain (inset with red border, Figure 3B) but within a closed (not the open) inter-domain pocket lined with amino acid side chains belonging to both the CARF and ADA domains. We wondered whether this mode of ATP binding was important for Cad1 activity, and therefore, we mutated the basic residues shown to interact with this molecule in the solved structures (inset, Figure 3B). In all cases, we made aspartate substitutions to change the charge of these residues and found that while K584D, R588D, K164D, and R162D did not affect Cad1 activation in staphylococci, K589D (within the deaminase domain), R161D, and K165D (within the CARF domain) prevented a complete growth arrest (Figure 3C).

We also solved the cryo-EM structures of cOA-bound Cad1. For both ligands, SECMALS analysis showed the presence of, as observed for apo-Cad1, a major hexameric fraction and minor dimeric and megadalton fractions (Figures S4D and S4E). We obtained the cA_4_-bound Cad1 structure from the hexameric fraction at 3.2 Å resolution in the presence of ATP (Figure 3D). In these conditions, Cad1 retains the trimer of dimers topology, with cA_4_ located within each dimeric CARF domain (Figures 3D and S4F). Superposition of the structure of this domain within the hexamer with that of the cA_4_-bound Cad1-CARF-His_6_ showed good structural correlation (root-mean-square deviation [RMSD] 2.8 Å), with a 3 Å upward shift of the ligand and a decrease in the angle between the C-terminal legs (57°) in the cryo-EM structure (Figure S4G). We believe this shift could be due to either the truncation of the full-length Cad1 protein in the case of the crystal structure, crystal-packing forces, or some combination of these factors.

**Figure 4.**
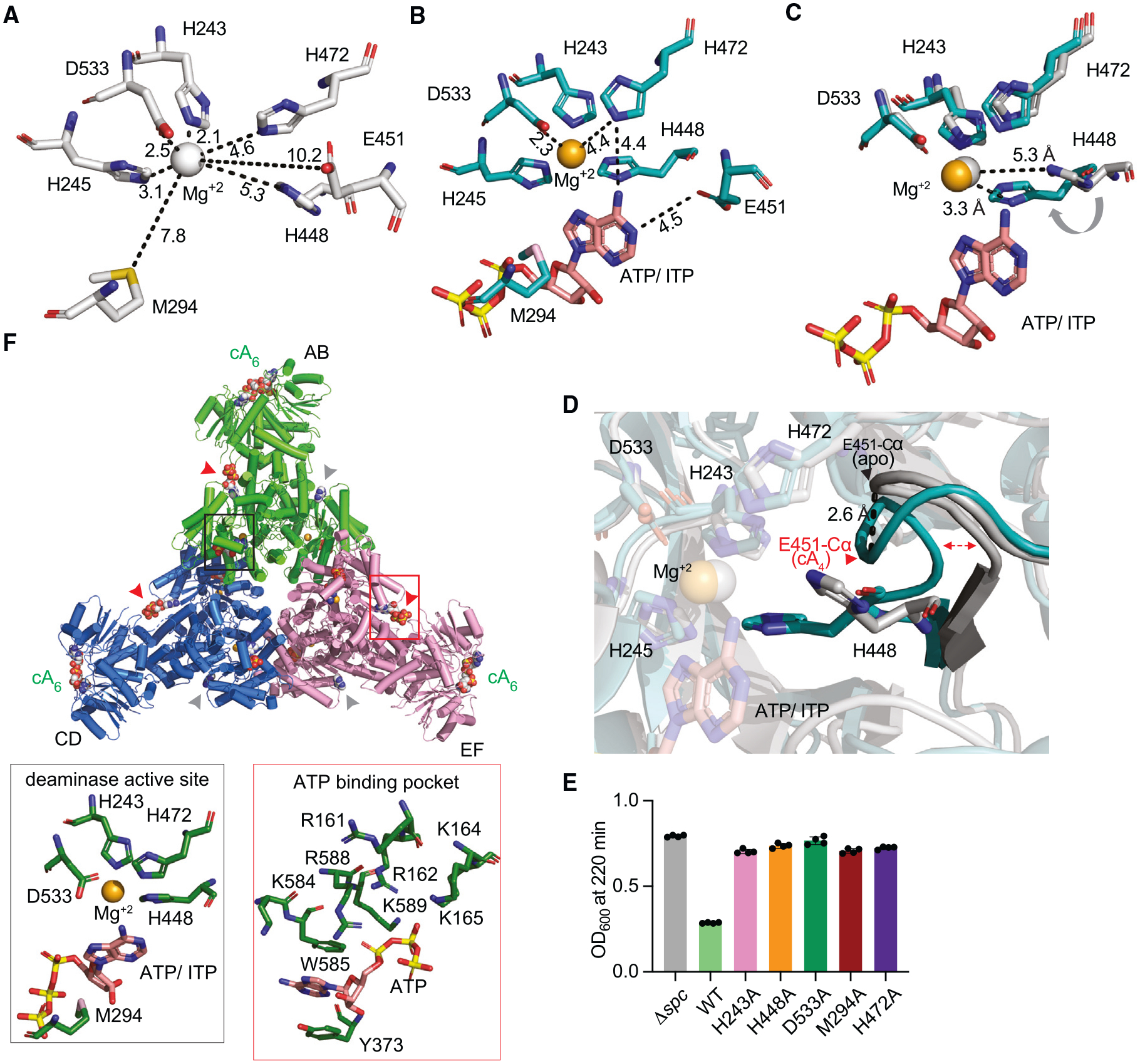
cOA-induced structural changes in the deaminase active site (A) Amino acid residues in the deaminase pocket of the apo-Cad1-ATP structure. Dotted lines indicate the distances (in Å) between side chains and the metal ion (modeled as Mg^+2^). (B) Same as (A) but for the deaminase pocket of cA_4_-Cad1-ATP. (C) Superimposition of the structures shown in (A) and (B). The shift in the H448 residue in the ATP-bound cA_4_-Cad1 structure is pointed out by a gray arrow. (D) Superposition of the deaminase pocket of the apo-Cad1-ATP structure (silver) and the cA_4_-Cad1-ATP structure (green). The shift in the loop containing H448 and E451 residues is shown by a red double arrow. The distance between the Cα atoms of E451 in the apo-Cad1-ATP structure (black arrowhead) and in the cA_4_-Cad1-ATP structure (red arrowhead) is marked by black dashed line. (E) Growth of staphylococci carrying pTarget and pCRISPR(Cad1) harboring the amino acid substitutions of the residues involved in ATP/ITP interactions at the deaminase site, measured as the OD_600_ value after 220 min of addition of aTc. Mean of four biological replicates, ±SEM, is reported. (F) Cryo-EM structure of hexameric cA_6_-bound Cad1 in the presence of ATP. cA_6_ was partly modeled within the CARF binding pocket due to the lack of density. ATP was modeled in three inter-domain binding sites (red border, with red arrowheads pointing at the ATP), and adenine was modeled at the other three sites (gray arrowheads). ATP/ITP and Mg^+2^ were modeled in four of the six deaminase pockets (black border inset) and only phosphate groups of ATP/ITP and Mg^+2^ in the other two due to lack of density. See also Figure S8.

Compared with the apo-Cad1 structure, the hexameric cA_4_-Cad1 complex displayed very minor changes (RMSD 0.6 Å, Figures S4H and S4I) with the notable exception that ATP density was observed in all six inter-domain pockets (Figure 3D). Close examination of the ATP-Cad1 interactions revealed that the phosphate groups were shifted deeper into the pocket, losing the contact with lysines and arginines from the loop of the CARF domain observed in the absence of the ligand (insets with red border, Figures 3B and 3D). Further, adenine bases shifted from *anti-* to *syn*-glycosidic bonds in the cA_4_-bound hexameric Cad1 (Figure S4J) but remained stacked between W585 and Y373 residues (Figure S4K). We also observed diffused density within all six deaminase pockets in the cA_4_-Cad1 complex, matching a metal divalent ion (which we modeled as Mg^+2^) and a nucleotide, which we were unable to determine whether it corresponded to ATP or to its deamination product ITP (insets with black border, Figure 3D). Close examination of the deaminase active site in the presence or absence of substrate (Figures 4A–4C) revealed that the positions of the metal ion and the H243, M294, H472, and D533 side chains remained unaltered. By contrast, H448 showed a shift toward the metal ion by 2 Å in the presence of the ligand (Figures 4C and 4D). To determine the importance of these residues for Cad1 activity, we mutated each of them to alanine and found all the substitutions abrogated Cad1-mediated cell toxicity, thus affecting Cad1’s ability to increase ITP levels *in vivo* (Figure 4E). Finally, we obtained the cA_6_-bound cryo-EM structure with a resolution of 3.6 Å, which also exhibited a trimer of dimers topology (Figure 4F). In contrast to cA_4_-bound Cad1, however, the tracing of both the ligand and ATP/ITP molecules was poor (Figure S5). Altogether, structural analysis of Cad1 revealed an unexpected hexameric topology capable of binding cOAs at the CARF domain and ATP molecules at both inter-domain and deaminase sites, with the amino acid chains lining these sites being important for Cad1 activation during the type III-A CRISPR-Cas response.

### Hexameric Cad1 converts ATP to ITP

**Figure 5.**
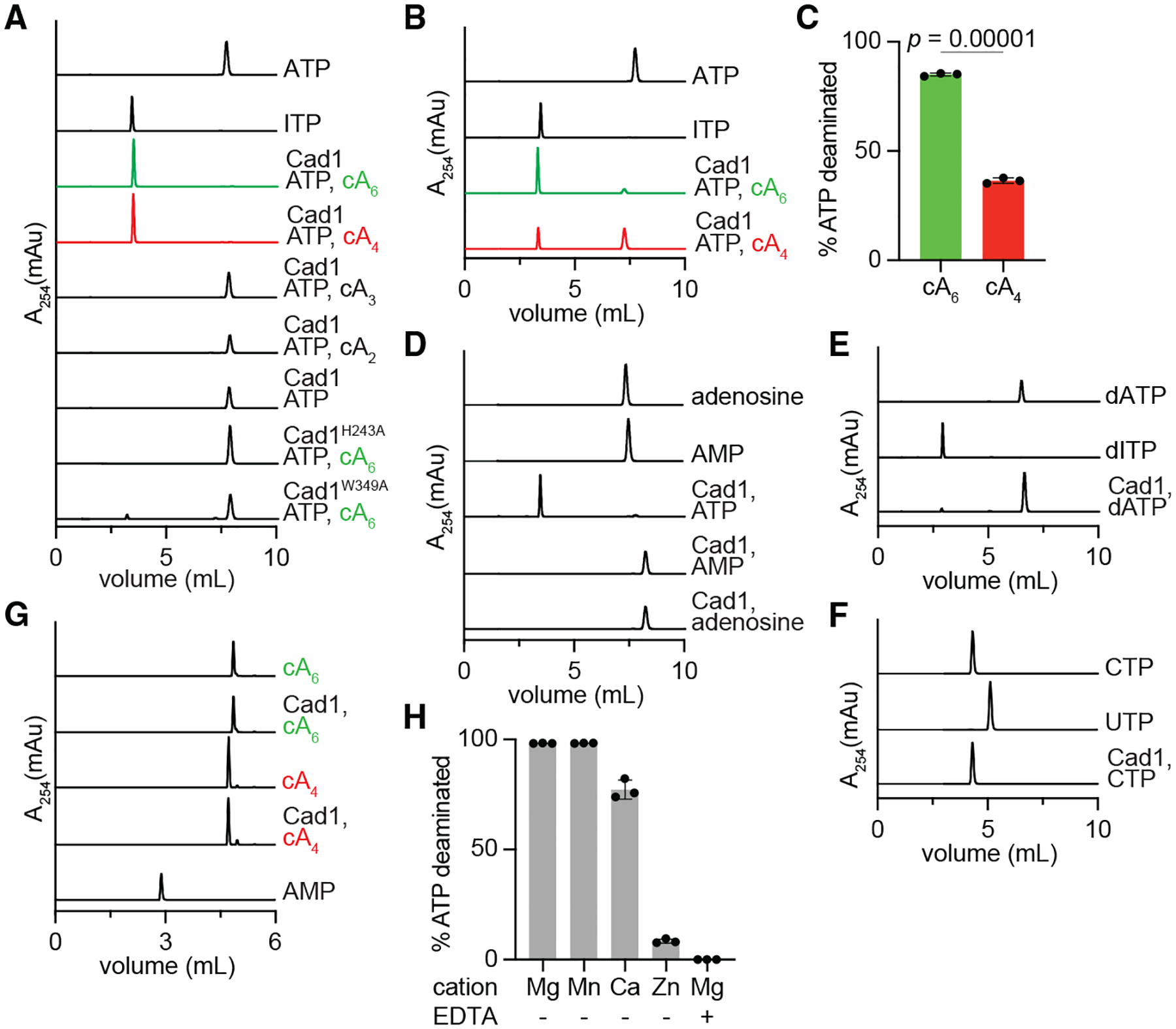
Cad1 converts ATP to ITP (A) HPLC analysis of Cad1 (wild-type and mutant versions; 2 μM) reaction products in the presence or absence of ATP (1 mM) and different cOAs (20 μM). Chromatograms of ATP and ITP are shown as standards. Reactions were performed in duplicate. (B) Same as (A) but in the presence of ATP and 100 nM of the indicated cOA. (C) Quantification of the product peaks obtained in (B) as percent of ATP substrate deaminated by Cad1. Reactions performed in triplicate, ±SEM, are reported. *p* values, obtained with a two-sided t test with Welch’s correction, are shown. (D) HPLC analysis of Cad1 (2 μM) reaction products in the presence adenosine, AMP or ATP (1 mM), and cA_6_ (20 μM). Chromatograms of adenosine and AMP are shown as standards. Reactions were performed in duplicate. (E) Same as (D) but using dATP as a substrate. (F) Same as (D) but using CTP as substrate. (G) HPLC chromatograms of Cad1 incubated with the indicated cOA at 500 μM. AMP is provided as a standard. Products of ring nuclease activity would be expected to run between cA_6_ and AMP. (H) Quantification of the percent of ATP substrate deaminated by Cad1 incubated with different divalent cations at a concentration of 1 mM except for Zn^2+^, which was incubated at a concentration of 100 μM to mitigate oxidation-induced aggregation of Cad1. A reaction using Mg^+2^ was performed in the presence of 3 mM EDTA. Reactions were performed in triplicate, and areas under the curve for ATP and ITP peaks were used to determine the % deamination. See also Figure S6.

To investigate the putative adenine deamination activity of Cad1, we incubated the purified hexameric protein (2 μM) with ATP (1 mM) and different cOAs (20 μM) and separated the reaction products using HPLC. We found that addition of cA_6_ and cA_4_, but not cA_2_ or cA_3_, led to the production of ITP (Figure 5A).Consistent with the observation that cA_6_ binds with more affinity than cA_4_ to the Cad1’s CARF domain, we observed approximately twice the amount of ATP deamination when we used a lower concentration of cOAs (100 nM, Figures 5B and 5C). We also tested activity toward other substrates such as adenosine and AMP (Figure 5D), dATP (Figure 5E), and CTP (Figure 5F); however, we were unable to detect any significant deamination of these substrates in the presence of cA_6_. Finally, because many CARF effectors have been found to cleave their ligands,^24^ we tested the ability of Cad1 to degrade cA_4_ and cA_6_. We incubated the hexameric fraction with cOAs at 37° for 16 h and separated the reaction products through HPLC (Figure 5G). We did not observe changes in the cOA signals, a result that suggests the Cad1 does not cleave its ligands.

Since the structural data revealed the presence of a metal cation in the deaminase site (Figures 3B, 3D, and 4A–4D), we used 1 mM MgCl_2_ in the above assays. To investigate in more detail the metal dependance of Cad1’s deamination reaction, we tested a panel of divalent cations that included Mg^+2^, Mn^+2^, Ca^+2^, and Zn^+2^ We observed a preference for Mg^+2^ or Mn^+2^ and weaker activity in the presence of Ca^+2^ or Zn^+2^, and that deamination was completely abrogated by the addition of EDTA (Figure 5H). This is a somewhat surprising result given that previously reported structures of ADAs from various species identified zinc as the metal cofactor in the deaminase pocket.^25,26^ We also tested the importance of H243, one of the residues that coordinates the metal ion (Figures 3B, 3D, and 4A–4D), which substitution for alanine abrogated Cad1 activity *in vivo* (Figure 4E), for ATP deamination *in vitro*. We found that cA_6_ was not able to activate ITP production in this mutant, confirming the role of H243 in catalysis (Figure 5A).

**Figure 6.**
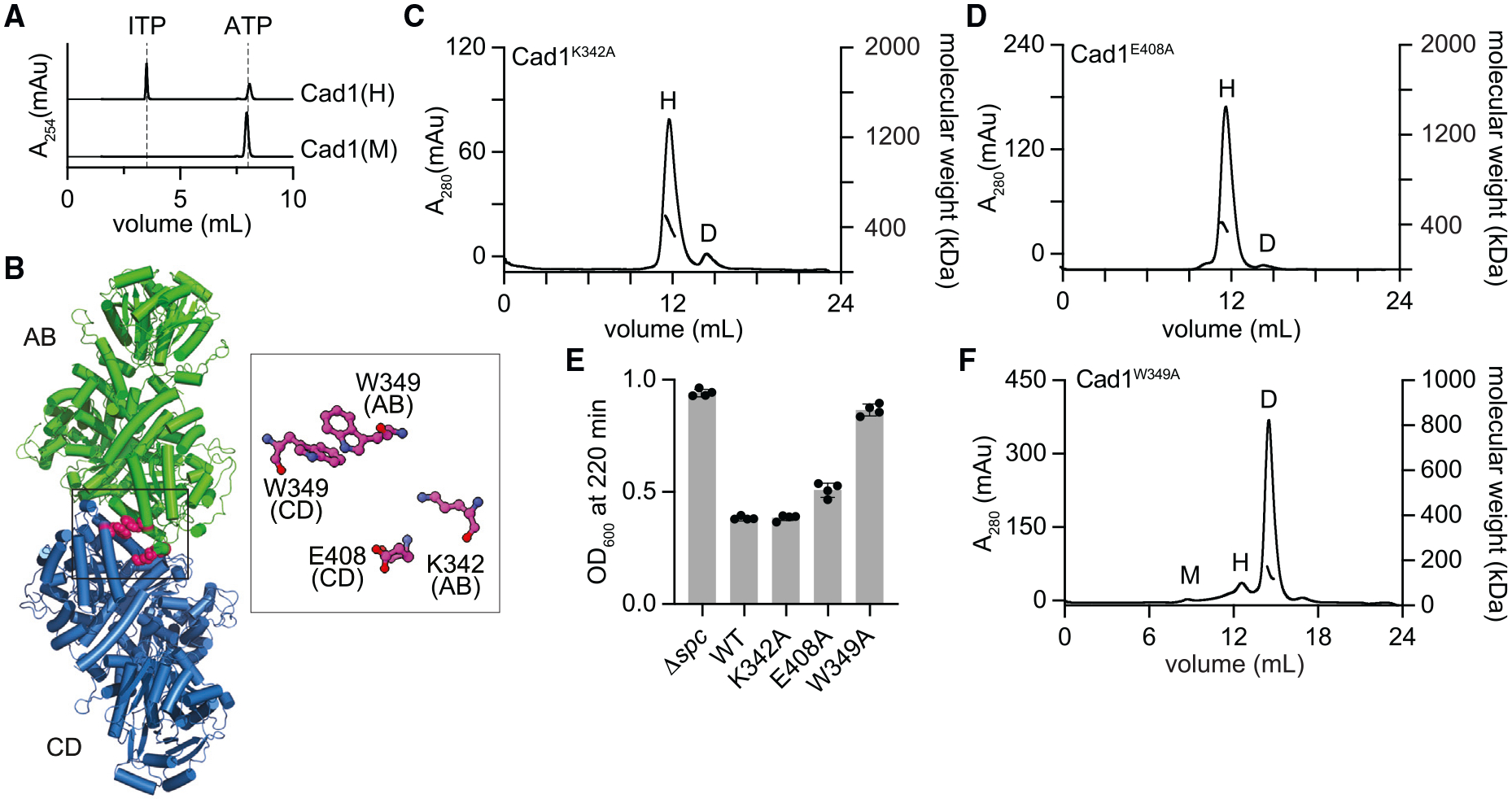
Oligomerization is required for Cad1 deamination (A) HPLC analysis of reactions products of incubation of different Cad1 fractions (H, hexameric; M, megadalton) in the presence of cA_6_ and ATP. Reactions were performed in duplicate. (B) Side view of the AB and CD dimers in the apo-Cad1 hexamer structure illustrating the residues present at the dimer-dimer interface. The residues mutated in this study are shown in the inset. (C) SECMALS analysis of hexameric Cad1^K342A^ (412 kDa ± 0.485%). (D) SECMALS analysis of hexameric Cad1^E408A^ (394 kDa ± 0.378%). (E) Growth of staphylococci carrying pTarget and pCRISPR(Cad1) harboring alanine substitutions of residues thought to be involved in the association of Cad1 dimers, measured as the OD_600_ value after 220 min of addition of aTc. Mean of four biological replicates, ±SEM, is reported. (F) SECMALS analysis of purified dimeric Cad1^W349A^ (139 kDa ± 0.518%).

Next, we investigated the contribution of the different oligomeric states to Cad1 ATP deamination activity. We determined that the megadalton fraction (Figures S4D and S4E) was unable to produce ITP in the presence of ATP, Mg^+2^, and cA_6_ (Figure 6A). We attempted to purify Cad1’s dimeric fraction (Figures S4D and S4E); however, we found that it eluted mainly as a hexamer when it was re-injected into the size-exclusion column (data not shown). Therefore, we decided to generate alanine substitutions of residues present at the dimer-dimer interface to prevent hexamer formation (Figure 6B). We found that the K342A and E408A mutations did not affect hexamerization (Figures 6C and 6D) or cOA-induced toxicity *in vivo* (Figure 6E). On the other hand, mutation of W349, which interacts with its counterpart in another Cad1 dimer via π-π stacking of aromatic rings (inset, Figure 6B), led to the detection of a stable dimeric complex by SECMALS (Figure 6F). When incubated with ATP, Mg^+2^, and cA_6_, however, this complex failed to produce ITP (Figure 5A), a defect that was reflected in lack of toxicity in cells (Figure 6E). Altogether, these data demonstrate that Cad1 deaminates ATP molecules to ITP through the formation of an hexameric complex that requires a Mg^+2^ or Mn^+2^ cofactor and an activating cA_4_ or cA_6_ ligand.

### RNA molecules are not affected by Cad1

Given that ADAs involved in phage defense have been reported to act on RNA molecules,^27^ we investigated whether Cad1 can modify transcripts as well. First, we incubated a 41-nt synthetic RNA substrate (Figure S6A) with Cad1 in the presence of cA_6_ and Mg^+2^. Reaction products were treated with P1 and calf intestinal alkaline phosphatase (CIP) nuclease to generate nucleosides for HPLC analysis, which did not detect any significant signal for modified nucleosides (Figure S6B). We also reasoned that Cad1 ATP deaminase activity could lead to misincorporation of ITP molecules into cellular transcripts. To test this, we extracted cellular RNA from staphylococci in which Cad1 was activated by the type III-A CRISPR-Cas response upon aTc addition, or control cells lacking *cad1*, and performed RNA sequencing (RNA-seq). We then mapped the sequencing reads to the genome of the host (*S. aureus* RN4420) to determine mutation frequencies, but we were unable to identify significant changes upon Cad1 activation (Figure S6C). These results suggest that Cad1 does not use RNA as substrates, nor that production of ITP during Cad1’s activation affects transcription fidelity.

### Cad1 provides antiviral immunity

**Figure 7.**
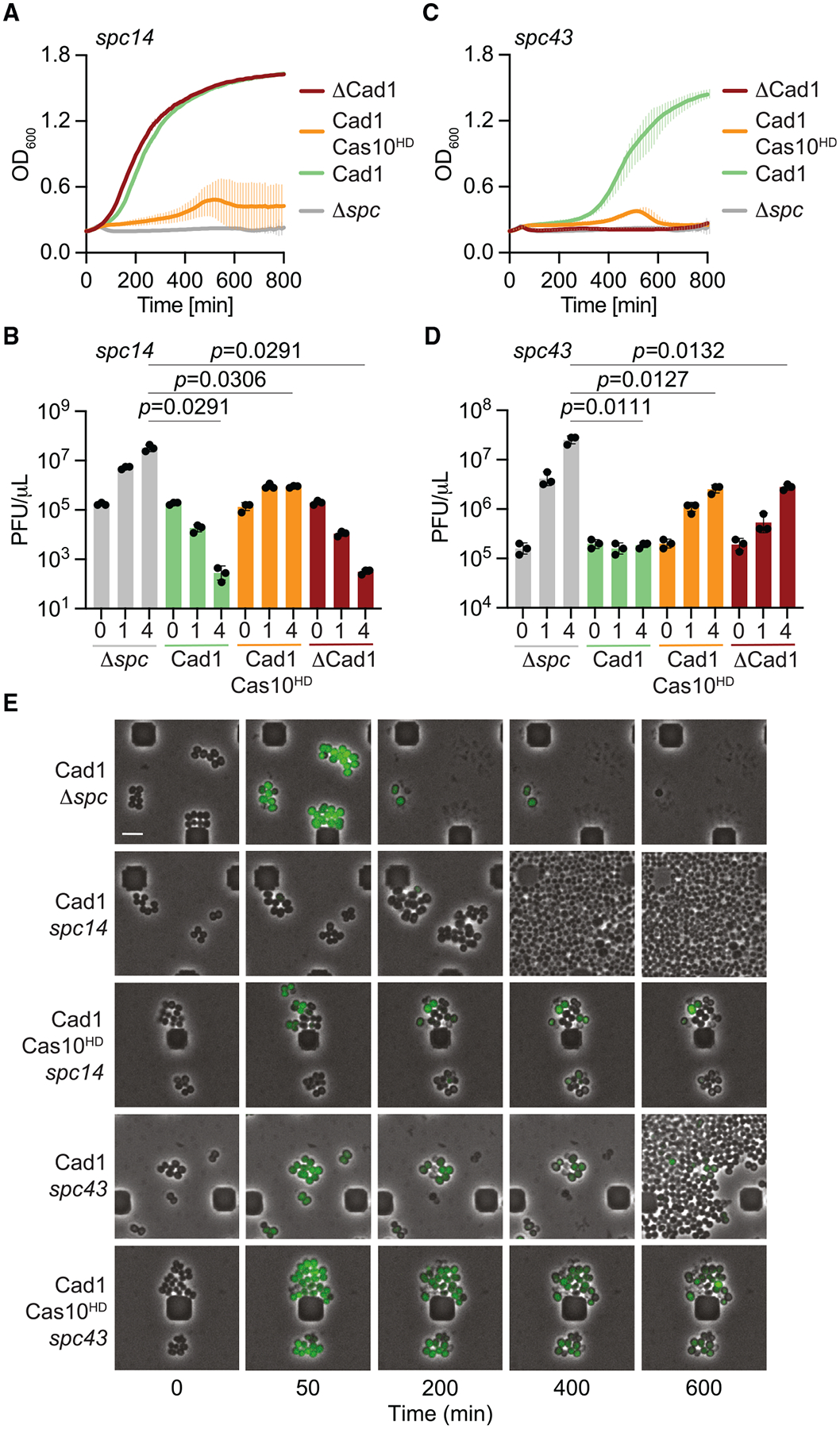
Cad1 provides antiviral immunity (A) Growth of staphylococci carrying different pCRISPR constructs programmed to target the *gp14* transcript of ΦNM1γ6-GFP, measured as OD_600_ after infection at an MOI of ~5. Mean of three biological triplicates, ±SEM, is reported. (B) Enumeration of plaque-forming units within staphylococcal cultures harboring different pCRISPR constructs programmed to target the *gp14* transcript, at the indicated times after infection with ΦNM1γ6-GFP at an MOI of ~1. Mean of three biological replicates, ±SEM, is reported. *p* values, obtained with a two-sided t test with Welch’s correction, are shown. (C) Same as (A) but targeting the *gp43* transcript. (D) Same as (B) but targeting the *gp43* transcript. (E) Time course microscopy of *S. aureus* harboring different pCRISPR constructs after infection with ΦNM1γ6-GFP. Images are representative of two biological replicates. Scale bar, 4 μM. See also Figure S7.

Finally, we tested the importance of Cad1 during the type III-A CRISPR-Cas response against phage infection. Given the different nature of this response depending on the timing of expression of the target RNA during the lytic cycle,^14^ we designed two spacers (*spc14* and *spc43*) producing crRNAs complementary to an early- and late-expressed transcript from the staphylococcal phage ΦNM1γ6-GFP (*gp14* and *gp43*, respectively; Figure S7A). This phage is a derivative of phage ΦNM1γ6^28^ that expresses green fluorescence protein (*gfp*) early during the lytic cycle and allows detection of infected staphylococci using fluorescence microscopy. In addition, since the type III-A defense in staphylococci relies on the function of both CARF effectors and the nuclease activity of Cas10,^12–14^ we tested different mutant type III-A systems with (1) *cad1* and wild-type *cas10* [pCRISPR(Cad1)], (2) with *cad1* but without the nuclease activity of *cas10* [pCRISPR (Cad1, Cas10^HD^)], (3) without *cad1* but with wild-type *cas10* [pCRISPR(ΔCad1)] as well as with a non-targeting spacer (Δ*spc*) as a negative (no immunity) control (Figure S1B). After infection at multiplicity of infection (MOI) ~5, we observed that when the target RNA is recognized early in the viral lytic cycle (*spc14*), Cas10 alone can provide defense to support the continued growth of staphylococci, measured as the OD_600_ of the infected culture (Figure 7A), in agreement with previous reports.^12,13^ By contrast, Cad1 alone provided a much weaker immunity. When we measured phage propagation through the enumeration of plaque-forming units (PFUs), we found that Cad1 was sufficient to halt the production of viral particles since it prevented the increase in PFU observed in the cultures without type III-A immunity (Δ*spc*) but was not able to reduce PFU counts as efficiently as Cas10 (Figure 7B), a result that corroborates the growth curves obtained after infection. When the type III-A response is delayed until late in the lytic cycle (*spc43*), both Cas10 and Cad1, but not each of these alone, are required to support the growth of the infected cells (Figure 7C) and reduce the PFU count (Figure 7D). We conclude that, as previously described for other CARF effectors, Cad1 is essential when the type III-A immune response is activated late in the viral lytic cycle when the nuclease activity of Cas10 is not sufficient to control the infection.

The results described above were corroborated and expanded using fluorescence microscopy of staphylococci treated with ΦNM1γ6-GFP, which enables the visualization of individual infected cells. In the absence of a targeting spacer that would activate the type III-A CRISPR-Cas response, cells turn green ~50 min post-infection and lyse shortly after (Figure 7E; Video S1). When both Cas10 and Cad1 are activated by an early expressed phage target transcript, cells continue growing after infection without any observable fluorescence, presumably due to the rapid clearance of the viral DNA by Cas10 ssDNase activity (staphylococci that do not express Cad1 and rely solely on Cas10 for immunity follow the same growth dynamics; Figure 7E; Video S2). By contrast, the majority of cells harboring pCRISPR(Cad1, Cas10^HD^) turned green and stopped proliferating (Figure 7E; Video S3), but did not lyse as in the case of the Δ*spc* control bacteria (Figure 7E; Video S1). This result indicates that Cad1 mediates a growth arrest when ITP production is triggered by phage infection, which cannot immediately halt the viral lytic cycle (it does not affect *gfp* expression) but eventually prevents full phage propagation (Figure 7B). In cultures harboring *spc43* in the presence of both Cas10 and Cad1 activities, infected staphylococci expressed GFP and entered a growth arrest, while some of the cells that did not emit green fluorescence (presumably not infected) were able to slowly resume growth (Figure 7E; Video S4). Therefore, when the type III-A immune response is triggered after the phage has entered the late stages of the lytic cycle, both Cad1 and Cas10 are required to stop viral spread to uninfected members of the population, leading to the survival of the bacterial culture. By contrast, cultures expressing Cad1 without the nuclease activity of Cas10 failed to regrow (Figure 7E; Video S5). Cells expressed GFP and stopped growing, with some undergoing lysis as well. In addition, phage replication led to a lower titer increase than that observed in the absence of immunity (Figure 7E) and produced smaller plaques on lawns of staphylococci expressing Cad1 (Figures S7B and S7C). Plaquing assays were also performed with the *Desulfomicrobiaceae* Cad1 homolog, and similar results were obtained (Figure S7D). These results suggest that ATP deamination alone when targeting a late-expressed transcript has a minor but noticeable effect on viral propagation. We also tested immunity against additional staphylococcal phages, ϕNM4γ4, ϕ80α*vir*, ϕJ1, and ϕJ4,^29^ by performing plaque assays on a lawn of cells carrying a type III-A CRISPR locus programmed with different spacers (Figure S7E) targeting these phages and with the *cas10*^HD^ mutation, to eliminate the immunity provided by the nuclease activity of the Cas10-Csm complex. We found a reduction of PFU in all cases (Figure S7F), a result that demonstrates that Cad1 provides immunity against a broad range of phages. Altogether, our data demonstrate an essential role for Cad1 during type III-A CRISPR-Cas immunity when the invading phage is recognized late in the infection cycle.

## DISCUSSION

CARF effectors have been shown previously to provide immunity via nucleic acid cleavage^13,15,17,22,30,31^ and transmembrane depolarization.^12^ Here, we present a new type of chemistry mediated by a CARF effector, deamination of ATP molecules to generate ITP. This reaction leads to a delay in the growth of staphylococci and to the survival of a population attacked by a phage that is recognized by the type III-A CRISPR-Cas system during the late stages of the lytic cycle, causing the abortion of the infection process and preventing viral spread. Although we do not know how the increase in ITP levels causes growth arrest in staphylococci, cells from organisms across different kingdoms of life are affected by the accumulation of this nucleotide, evidenced by the toxic effects of loss of ITPase enzymes.^32–36^ It is conceivable that ITP competes for binding sites typically occupied by ATP in proteins that are critical for normal cellular function; it is also possible that high levels of ITP interfere with phage DNA replication. Importantly, another anti-phage defense system, restriction by an ADA acting on RNA (RADAR) also uses this strategy.^27,37,38^ While one report showed that deamination leads to the accumulation of mutations in cellular and phage transcripts,^27^ two additional studies demonstrated that RADAR deaminates both dATP and ATP, leading to a toxic increment of (d)ITP levels in the infected cells.^37,38^ While Cad1 forms a hexameric complex that is activated by cA_4_ and cA_6_ molecules upon RNA-guided recognition of the invader, RADAR forms a dodecameric, hollow complex composed of two different subunits, an ATP deaminase effector and a AAA+ ATPase, which activation mechanism during infection remains unknown.^37,38^ We believe that these differences reflect a divergent evolution pathway for adenosine deamination as a mechanism of defense in the context of the phage-host arms race. Given the vast diversity of phage predators, hosts are forced to evolve different strategies to sense infection to activate adenosine deamination. Deamination of dCTP to dUTP is also known to provide immunity against phage infection.^39,40^ These deaminase systems deplete dCTP from the nucleotide pool, causing cell toxicity to prevent viral DNA replication within the infected host.

Several unanticipated aspects emerged from the structural characterization of Cad1. First, two distinct cOAs, cA_4_ and cA_6_, were found to bind to the CARF domain. Second, while the deaminase pocket was empty in apo-Cad1 hexamer in the presence of ATP, it was occupied by ATP/ITP in the ligand-bound Cad1 hexamer complex. Third, we unexpectedly found ATPs bound in inter-domain pockets between the CARF and deaminase domains, with three ATPs (*anti*-glycosidic bonds) in apo-Cad1 hexamer increasing to six ATPs (*syn*-glycosidic bonds) in the ligand-containing complex (Figures 3B, 3D, S4J, and S4K). This observation suggests that ATP binding to non-deaminase inter-domain pockets impacts the activation of the deaminase active site. Fourth, though the apo-Cad1 hexamer and cA_4_-Cad1 hexamer complex superpositioned quite well, we did observe a specific catalytic histidine (His448) shift by 2 Å toward the metal ion (modeled as Mg^2+^) in the deaminase pocket on complex formation, with this movement likely to contribute to the generation of a catalytically competent site for ATP to ITP conversion (Figures 4A–4D). By comparing Cad1’s deaminase active site with that of *Mus Musculus* ADA bound to 1-deazaadenosine^26,41^ (Figure S8A), we propose a catalytic mechanism in which a histidine residue (H472 or H448) activates the OH group of a water molecule (possibly coordinated by residue D533 and the Mg^+2^ ion, but not observable in the cryo-EM structure of the complex at 3.2 Å resolution) that makes a nucleophilic attack on the C6 carbon of the adenine base, with E451 acting as the receptor for the NH leaving group (Figures S8B–S8E). The structures of individual ATP-bound Cad1 dimers (in a hexameric context) in the absence and presence of cA_4_ did not exhibit major conformational changes between states (Figures S4H and S4I). The main difference is in ATP binding. While three ATP (*anti*-alignment) molecules are present in three interdomain pockets and not in deaminase pockets in the absence of cA_4_ (Figure 3B), six ATP (*syn*-alignment) molecules are bound in each interdomain pockets, and six ATPs/ITPs are bound in the deaminase pockets in the presence of the ligand (Figure 3D). Somehow, in a process that we cannot currently explain, the switch from *anti-* to *syn*-alignments of the ATPs bound in the interdomain pockets (Figures S4J and S4K) and/or the occupation by ATP/ITP of the deaminase pockets contribute to the shift of His448 on cA4 addition (Figures 4C and 4D) to generate a catalytically competent active site.

Finally, we observed that the hexameric, but not the dimeric, form of Cad1 is active, *in vitro* and *in vivo* (Figures 5A and 6E). This suggests hexamer disassociation as a potential autoregulation mechanism for Cad1 that could mitigate the cellular toxicity caused by ITP accumulation, maintain a growth arrest that does not turn into cell death, and/or enable the host to regain the ability to proliferate after phage infection. While the triggering of a growth arrest in the host is a common strategy of all CARF effectors,^12,13,15,17,22,30,31^ we believe that Cad1 introduces a new mode of defense for CRISPR-Cas systems that couples nucleotide deamination activity to a sequence-specific, RNA-guided adaptive immune response.

### Limitations of the study

In our experimental system, we heterologously expressed Cad1 homologs from either *Bacteriodales* or *Desulfomicrobiaceae* bacteria in *S. aureus* in the context of the staphylococcal type III-A CRISPR-Cas immune response. Therefore, it is possible that some of functions we describe for Cad1 could be different in the native hosts, which do not share the same physiology nor the infecting viruses with *S. aureus*.

Even though our study explains the mechanism of ATP deamination by the catalytic residues lining the deaminase pocket of Cad1, we do not yet have a mechanistic explanation as to what subtle conformational changes contribute to the shift of the His448 residue toward the metal to generate a catalytically competent pocket upon ligand binding. In addition, though we obtained the structural details of the interaction between cA_4_ and the CARF domain in hexameric Cad1, we were unable to show the cA_6_ density in the hexameric complex formed by this ligand. Unlike the use of a short incubation time used prior to formation of cA_4_-bound Cad1 grids, overnight incubation was used prior to formation of cA_6_-bound Cad1 grids, which may have led to the disintegration of the complex in the latter case.

## RESOURCE AVAILABILITY

### Lead contact

Further information and requests for resources and reagents should be directed to and will be fulfilled by the lead contact, Luciano A. Marraffini (marraffini@rockefeller.edu).

### Materials availability

Plasmids generated in this study are available from the lead contact upon request.

### Data and code availability

- All standardized datasets are available as follows: cryo-EM maps have been deposited in the Electron Microscopy Data Bank (EMDB) under the accession codes EMDB: EMD-45241 (apo-Cad1), EMD-45244 (ATP-Cad1-symmetric), EMD-45245 (ATP-Cad1-asymmetric), EMD-45277 (ATP-Cad1-cA4), and EMD-45466 (ATP-Cad1-cA6). The corresponding atomic coordinates of the cryo-EM structures have been deposited in the Protein Data Bank (PDB) under the accession codes PDB: 9C67 (apo-Cad1), 9C6C (ATP-Cad1-symmetric), 9C6F (ATP-Cad1-asymmetric), 9C77 (ATP-Cad1-cA4), and 9CDB (ATP-Cad1-cA6). Atomic coordinates of the X-ray structures have been deposited in the PDB under the accession codes PDB: 9C6A (apo-Cad1-CARF), 9C68 (Cad1-CARF + cA6), and 9C69 (Cad1-CARF + cA4). Cad1 RNA-seq raw reads used for mutation frequency calculation are deposited in NCBI’s SRA database with BioProject: PRJNA1152273.
- The script used to calculate normalized mutation frequencies has been uploaded to https://github.com/Marraffini-Lab/Baca_and_Majumder_et_al_2024. All other analyses were performed with the reported programs and packages in the key resources table.
- Any additional information required to reanalyze the data reported in this paper is available from the lead contact upon request.

## STAR★METHODS

### EXPERIMENTAL MODEL AND STUDY PARTICIPANT DETAILS

#### Bacterial strains and culture conditions

The *Escherichia coli* Rosetta 2 (DE3) cells used for protein purification were grown in Luria broth (LB) with antibiotics at 37°C for growth phases and 18°C for protein expression. Staphylococcus aureus RN4220 strains were grown in Brain Heart Infusion (BHI) broth at 37°C for all experiments.

### METHOD DETAILS

#### Sequence alignments

Alignments and calculation of sequence identity and similarity (Figure S1C) were determined using EMBOSS Needle pairwise sequence alignment.^50^

#### Bacterial growth

*Staphylococcus aureus* strain RN4220^23^ was grown in brain heart infusion (BHI) medium at 37°C, supplemented with chloramphenicol at 10 μg ml−1 for maintaining pCRISPR, and erythromycin at 10 μg ml−1 for maintaining pTarget. 5 μM CaCl_2_ was supplemented in phage experiments unless indicated otherwise.

#### Plasmid construction

The plasmids, oligonucleotides and cloning strategies for generating the plasmids used in this study are detailed in Data S1. For obtaining the coding sequence of Cad1, the amino acid sequence of NCBI GenBank reference sequence HAJ98955.1 from Bacteriodales bacterium was synthesized by Genewiz.

#### Growth curves

For *in vivo* Cad1 toxicity induction, biological replicates of RN4220 overnight cultures containing pTarget and pCRISPR are diluted 1:100, outgrown for about an hour and normalized for optical density. Cells are then seeded in a 96-well plate. To induce targeting, 125 ng ml^−1^ of aTc is added to the appropriate wells. Absorbance at 600 nm is then measured every 10 min by a microplate reader (TECAN Infinite 200 PRO).

For *in vivo* antiphage immunity, cells containing various pCRISPRs were launched in triplicate overnight, diluted 1:100, outgrown for about an hour and normalized for optical density. Cells were seeded into a 96-well plate. ΦNM1γ6-GFP was added at the specified MOIs, and optical density measurements were taken every 10 min.

#### Cad1 toxicity assay

To measure the effect of Cad1 activity on *S. aureus* viability over time, colonies of *S. aureus* containing pTarget and the specified pCRISPR were launched in liquid culture overnight in triplicate. The next day, cells were diluted 1:100 and grown out for about 1 h and normalized for optical density. One aliquot was taken from each culture, and then aTc was added to induce CRISPR targeting and Cad1 activity. At each time point, cell aliquots were removed, centrifuged and resuspended in medium lacking aTc, and serial dilutions were plated on solid BHI agar plates with or without aTc. All viable cells should grow on the solid agar plates, but only targeting escapers (cells that recover owing to mutations in pTarget or pCRISPR) should form colony-forming units on plates with aTc.

#### Phage plaquing and enumeration of phage plaques

To obtain plaque-forming-unit counts over time from cultures infected with phage, *S. aureus* cultures containing various pCRISPRs were launched overnight, diluted 1:100 and outgrown for about one hour. Cells in media supplemented with 5 mM CalCl_2_ were then infected with phage ΦNM1γ6-GFP at an MOI of 1, and an aliquot was taken shortly after to obtain plaque-forming units at time 0. The cultures were then incubated further, with aliquots taken at one and four hours. For phage plaquing assays, indicated phage stocks were plaqued on lawns of *S. aureus* containing the indicated constructs, or cells lacking an introduced pCRISPR plasmid to measure PFU changes over time, with 10-fold serial dilutions for every spot in a lane.

#### Cad1 toxicity time-course microscopy

To monitor the effects of Cad1 toxicity dynamics in greater detail, colonies of *S. aureus* containing pTarget and the specified pCRISPRs were launched in liquid culture overnight. The next day, cells were diluted 1:200 and were loaded into a CellASIC^®^ ONIX microfluidic plate along with media containing plain BHI and media containing BHI spiked with 125 ng ml^−1^ aTc. The plate was sealed and connected to a CellASIC^®^ ONIX2 Microfluidic System for microfluidic control of cells and media. Cells were incubated in the plate for 1 h at 37°C using a Tokai HIT thermal box (Zeiss) until they were loaded onto the imaging chamber. Cells were imaged with phase contrast every 2 min for 8 h. Plain BHI was flowed over cells for the first hour and 15 min followed by BHI spiked with aTc for the remaining 5 h and 45 min. Imaging was performed in a Nikon Eclipse Ti2 motorized microscope with Perfect Focus System using a CFI60 Plan Apochromat Lambda Phase Contrast DM 100x Oil Immersion objective lens (Nikon) with a Zyla 4.2 sCMOS (Andor) camera (65 nm pixels). We used a SOLA Light Engine (Lumencor) as a laser source with laser power set to 20% with an exposure time of 10 ms. All media was flown over cells with a constant pressure of 13.8 kPa.

#### Time-course fluorescence microscopy of phage-infected cultures

To visualize the dynamics of phage infection and immunity provided by Cad1, colonies of *S. aureus* containing various pCRISPRs with spacers programmed to target specified ORFs in ΦNM1γ6-GFP were launched in liquid culture overnight. The next day, cells were diluted 1:200 and were loaded into a CellASIC^®^ ONIX microfluidic plate along with media containing plain BHI supplemented with 2.5 mM CaCl_2_ with and without ΦNM1γ6-GFP at a titer of 2.0 × 10^7^ PFUs ml^−1^. The plate was sealed and connected to a CellASIC^®^ ONIX2 Microfluidic System for microfluidic control of cells and media. Cells were incubated in the plate for one hour at 37°C using a Tokai HIT thermal box (Zeiss) until they were loaded onto the imaging chamber. Cells were imaged with phase contrast and in a GFP fluorescence channel every 2 min for 17 h. For the first hour, BHI supplemented with CaCl_2_ was flowed over cells followed by 15 min of phage flowed over. Finally, BHI supplemented with CaCl_2_ was flowed over for the remaining 15 h and 45 min. The same phase contrast settings used in the Cad1 toxicity microscopy were used in these experiments, however, the GFP channel was measured with a C-FL GFP HC HISN Zero Shift filer (Excitation: 470/40 nm (450–490 nm), Emission: 525/50 nm (500–550 nm), Dichroic Mirror: 495 nm) (Nikon). GFP channel imaging was performed with the SOLA Light Engine set to 2% laser power with a 200-ms exposure time. All media was flown over cells with a constant pressure of 13.8 kPa.

#### Construction of the Cad1 homolog phylogenetic tree

Homologs of Cad1 were collected by performing a Blastp search with default parameters from our original Cad1 protein sequence. Homologs sequences were collected and filtered for redundancy by removing homologs that have over 95% sequence identity to another homolog. Additionally, only homologs with a sequence length between 550 and 650 were collected (original Cad1 is 600 residues in length) for downstream analysis to remove homologs that do not contain either a CARF or adenosine deaminase domain. The remaining 88 homologs were then aligned using Clustal Omega with default parameters. The resulting alignment file was used to generate a phylogenetic tree using the Geneious tree builder tool. The resulting tree was then visualized with iTOL. The tree was annotated with the phylum of bacterium each homolog comes from along with CRISPR associations. CRISPR associations were determined using the CRISPRCasTyper tool^43^ against all genomes containing the Cad1 homologs. This was also used to calculate Type III CRISPR associations in Figure 1C.

#### In vitro deamination reactions

Deamination reactions were performed in *in vitro* by incubating substrates at 37°for 2 h unless otherwise indicated. Reaction substrates were supplied at 1 mM final concentrations (except for the RNA substrate which was supplied at a concentration of 20 mM) with Cad1 at 2 mM. Additionally, 1 mM of the indicated divalent cation cofactors and 20 mM of the indicated cOA activators were added to reaction mixes. All reactions were performed in 25 mM HEPES pH 7.5, 100 mM NaCl, 2 mM bME, and 5% glycerol. After incubations at 37°, reactions were then heated to 90° for 3 min to quench reactions and then cooled to 4° before removing from thermal blocks. Cooled reactions were removed and then supplemented with NEB quick CIP to dephosphorylate reaction products for subsequent HPLC analysis. For RNA substrates, quick CIP in addition to P1 nuclease was used to generate monophosphates that could then become dephosphorylated for HPLC analysis. Reactions were then incubated at 37° for an additional hour with the same heat inactivation treatment. Finally, reactions were diluted with nuclease-free water before being filtered with Amicon^®^ Ultra Centrifugal Filter, 3 kDa MWCO filters to remove proteins before analysis. 10 mL of final clean reaction products were then injected onto an Agilent Bonus-RP, 4.6 × 150 mm, 3.5 um Rapid Res. C18 column held at 40° at a flow rate of 1 mL per min with a constant mobile phase of 97% monobasic sodium phosphate pH 6.8 and 3% acetonitrile. Chromatograms were collected by monitoring absorbance at 254 nm.

#### In vitro ring nuclease reactions

To monitor potential ring nuclease activity by the CARF domain of Cad1, we incubated 500 mM cA_6_ or cA_4_ with 2 mM full length Cad1 or cOA alone in the same reaction buffer mentioned previously. Reactions were carried out at 37° for 16 h before filtering the reaction products and collecting chromatograms. Chromatograms were collected with a mobile phase of 100/0% A/B for 2 min followed by 95/5% A/B in two minutes, then 80/20% A/B in 1 and a half min, 75/25% A/B in 30 s, and 100/0% A/B in 2 min. Buffer A is composed of 60 mM dipotassium hydrogen phosphate and 40 mM potassium dihydrogen phosphate at pH 7 and Buffer B is pure acetonitrile.

#### Quantification of nucleotides from lysates using HPLC-coupled high-resolution mass spectrometry

Indicated strains were grown to an OD600 of 0.3. At this point 100 mL of these cultures were pelleted, resuspended in remaining supernatant and were subjected to our extraction method immediately. The remaining cells in culture were treated with 125 ng per mL aTc for 15 minutes before pelleting and collection. Resuspended cells were then treated with 80% ethanol supplemented with 4 mM EDTA, vortexed vigorously, and subjected to heating at 85° with shaking for 3 min followed by cooling to room temperature. The lysates were then spun down at 13,000 rpm on a tabletop centrifuge and supernatants were removed for further preparation. About 600 uL of these supernatants were dried under blowing air at room temperature for 2 h. The remaining extracts were reconstituted to 100 uL with 10 mM ammonium bicarbonate solution (pH 7) and run through 10 kDa filters at 14,000 x g, 4°C for 30 min. The filtrates were analyzed on Sciex X500 QTOF mass spectrometer with electrospray ionization in the negative mode. Samples were delivered by ExionLC AC system equipped with SeQuant^®^ ZIC^®^-HILIC (3.5 μm, 100Å_)_ 50 × 2.1 mm column that was held at 40°C during analysis. Solvent A was water with 50 mM ammonium acetate (pH 6.9) and solvent B was acetonitrile. The flow rate was 0.3 ml/min. For each 15-min run, 1 uL of sample was injected and separated with the following gradient: 74% B for 10 min, to 50% B in 0.1 min, 50% B for 0.9 min, back to 74% B in 0.1 min, 74% B for 4.9 min. Column eluate between 1.5 min and 11 min was directed to MS for analysis using the following MS method. The following parameters were applied for the ion source: curtain gas of 30 psi, ion source gas 1 of 50 psi, ion source gas 2 of 50 psi, temperature of 400°C, spray voltage of −4500 V. For TOFMS, m/z between 100–510 was scanned with a declustering potential of −80°C, collision energy of −10 V, collision gas of 7 psi, and accumulation time of 0.25 s. For TOFMSMS, SWATH (data independent acquisition) was applied with two TOFMS precursor ranges (505.5 Da to 506.5 Da for ATP and 506.5 to 507.5 Da for ITP) and following parameters: scan range of m/z 50–510, declustering potential of −80 V, collision energy of −35 V with 15 V spread, accumulation time of 0.1 s. All LC-MS and LC-MS/MS data were collected and viewed on the software Sciex OS. TOFMSMS data were used to confirm the identities of the captured exact m/z in comparison to ATP and ITP standards. Exact m/z (505.989 ± 0.01 Da for ATP, 506.975 ± 0.01 Da for ITP) intensities were extracted from LC-MS data to generate extracted ion chromatographs that were analyzed on Microsoft Excel to calculate area under curves based on the trapezoidal rule. Area under curves were used to represent the relative amount of ATP and ITP in each sample.

#### RNA extraction, sequencing, and mutation analysis

Cells containing the indicated pCRISPR strains with pTarget were grown to an OD600 of 0.3. aTc was added to cultures at a concentration of 125 ng per mL and incubated for 45 min. Cells were then pelleted, and RNA was extracted from cells using a Zymo Quick-RNA miniprep plus kit. Extracted RNA was then sent to SeqCenter for Illumina RNA sequencing with rRNA depletion. Samples were DNAse treated with Invitrogen DNAse (RNAse free). Library preparation was performed using Illumina’s Stranded Total RNA Prep Ligation with Ribo-Zero Plus kit and 10 bp unique dual indices (UDI). Sequencing was done on a NovaSeq X Plus, producing paired end 150 bp reads. Demultiplexing, quality control, and adapter trimming was performed with bcl-convert (v4.2.4). Read mapping was performed with HISAT2^46^ with default parameters with ‘—very-sensitive’. To quantify the frequency of nucleotide mutations within RNA sequencing datasets, .bam files were processed with REDItools V2^48^ to generate base conversion rates across reads. Datasets were then analyzed with a custom python script to calculate normalized mutation frequencies. For each position within the genome, the script calculated the mutation count for each possible base pair substitution. These mutation counts were then adjusted by multiplying by the coverage at the respective position and dividing by the total number of reads in the dataset. Subsequently, the normalized mutation frequencies for each type of base substitution were aggregated across the entire dataset to provide a comprehensive view of the mutation landscape. The results for each technical replicate were presented separately to preserve the granularity of the data and allow for analysis of mutation patterns across datasets.

#### Heterologous expression and purification of Cad1 protein

The His_6_-Cad1 construct, the His_6_-Cad1 mutants and His_6_-Cad1-CARF construct were transformed into *E. coli* Rosetta^™^ 2 (DE3) cells (Novagen). The cells were grown at 37° in Lysogeny broth (LB) media till the OD_600nm_ reached to 0.6–0.8 and then the cells were induced with 0.5 mM isopropyl β-D-1-thiogalactopyranoside (IPTG) and grown for another 14 h at 16°. The cells were harvested by centrifugation at 4000 r.p.m. for 15 min and resuspended in the lysis buffer (25 mM Hepes pH 8, 500 mM NaCl, 2 mM β-Mercaptoethanol and 5 % glycerol) supplemented with cOmplete mini, EDTA-free protease inhibitor tablets (Sigma). The resuspended cells were lysed by sonication and cell lysates were cleared from unlysed cell debris by centrifugation at 22000 r.p.m at 4° for 1 h. The supernatant was loaded to pre-equilibrated 5 ml HisTrap column (Cytiva). The column was washed extensively by the lysis buffer supplemented with 40 mM imidazole. The protein was eluted using the lysis buffer supplemented with 300 mM imidazole. The eluted protein was further purified by size-exclusion chromatography using Superdex 200 10/300-increase column (data not shown) pre-equilibrated in buffer A (25 mM Hepes pH 8, 200 mM NaCl, 2 mM β-Mercaptoethanol and 5 % glycerol).

#### Cad1 copurification of cOA products from cell lysates

To determine whether Cad1 bound cOA products produced from our CRISPR system in cells, 8 L of staphylococcal cells containing both pCRISPR with a hexahistidine-tagged Cad1 with a dead Cas10 HD domain background and pTarget were grew of an OD_600_ of 0.4. Cells were then induced with 100 ng/mL aTc to activate the production of cOAs within these cells for 30 min. Cells were then pelleted and lysed with lysostaphin and were purified with the same purification scheme described above except for the buffers containing 200 mM NaCl for all the steps in the purification process. The resulting pure protein was concentrated to 4 mg/mL, heat denatured at 85° to release any potential cofactors, and was filtered through a 10 kDa MWCO filter. Flow through from these filters were injected onto our C18 column for HPLC analysis with the same method used in our *in vitro* ring nuclease reaction analysis. 100 mM cA4 and cA6 standards that also underwent heat treatment and filtering were also used as references.

#### Sample preparation for SECMALS analysis to determine the oligomeric states of Cad1 protein under varying conditions

The experiment was performed with 20 μM of apo-Cad1 protein and the control apo-Cad1 run was performed at 0-h incubation using Superdex 200 increase 10/300 GL column (data not shown). Further, the same amount of apo-Cad1 protein was mixed with cA_4_/cA_6_, ATP and MgCl_2_ in 1:5:50:50 (Cad1-cA_4_/cA_6_-ATP-MgCl_2_) molar ratio and diluted to 5 ml of buffer A and incubated at 37°C for 4 h. The apo-Cad1 control sample was prepared similarly without supplementing it with cA_4_/cA_6_, ATP and MgCl_2_. The reaction mixture was concentrated to 500 μl using 50 kDa cut-off concentrator (Millipore Sigma) by centrifugation at 3500 rpm for 6 min. The concentrated reactions were loaded to the sizing column for the experiment and the SECMALS analysis was performed. After each run the sample was collected from the peaks correspond to the megadalton (M), hexameric (H), the dimeric (D) species and the unbound cA_4_/cA_6_ and ATP. All the collected fractions were pulled and incubated at 37°C for 14 h without any further addition of cA_4_/cA_6_ and ATP. The reactions were concentrated to 500 μl using 50 kDa cut-off concentrator using the same method as mentioned before. The reactions were loaded to the size column for the SECMALS analysis (the analyzed data are presented in Figures S4A, S4D, and S4E).

#### SECMALS analysis

The oligomeric states of the full-length Cad1 protein, its mutants and Cad1-CARF protein were determined by SECMALS analysis. The experiments were performed with AKTA-Pure UV detector connected to SECMALS instrument which has multi-angle light scattering detectors and refractive index detectors (Wyatt). The pure protein was loaded into Superdex 200 10/300-increase column pre-equilibrated with buffer A (25 mM Hepes pH 8, 200 mM NaCl, 2 mM β-mercaptoethanol and 5% glycerol). ASTRA 6 software was used for data analysis. The UV signal of the UV detector of the AKTA-Pure instrument was converted to the analogue signal with a conversion factor of 1,000 mAU = 1 V. For the analysis the refractive index increment (dn/dc) value was 0.185 ml g−1 for Cad1 protein, it’s mutants and Cad1-CARF protein.

#### Isothermal titration calorimetry (ITC) binding studies

The binding studies by ITC were performed using 10 μM apo-Cad1-CARF (1–185) protein titrated with 100 μM of cA_4_/cA_6_. Both the protein and the cyclic oligoadenylate ligands were present in the buffer (buffer A- 25 mM Hepes pH 8, 200 mM NaCl, 2 mM β-mercaptoethanol and 5% glycerol). The experiments were performed with MicroCal PEAQ-ITC (Malvern) at 25°C temperature. The titrations were performed with a total of 19 injections with 0.4 μl volume of the first injection (0.8 s duration) and the rest 18 injections (4 s duration) were of 2 μl. The spacing between each injection was 150 s and stirring rate was 750 rpm. For the data analysis and the *K*_d_ value estimation MicroCal PEAQ-ITC Analysis Software (Malvern) was used. The estimated *K*_d_ values are 30 nM ± 6 nM and 735 nM ± 215 nM for Cad1-CARF(1–185)-cA_6_ and Cad1-CARF(1–185)-cA_4_ respectively. In case of Cad1-CARF(1–185)-cA_6_ binding experiment the ΔH (kcal/mol), ΔG (kcal/mol) and -TΔS (kcal/mol) values are 29.3±0.5, −10.3 and −39.5 respectively. In case of Cad1-CARF(1–185)-cA_4_ binding experiment the DH (kcal/mol), DG (kcal/mol) and -TDS (kcal/mol) values are 22.3 ± 1.8, −8.37 and −30.7 respectively. The Cad1-CARF (1–185)-cA_6_ binding studies were repeated 3 times and The Cad1-CARF (1–185)-cA_4_ binding studies were repeated 2 times. The FitX and FitY values of the analyzed data were represented using GraphPadPrism version 9.4.0.

#### Protein crystallization

The purified His_6_-tagged apo-Cad1-CARF (1–185) was concentrated to 15 mg/ml in buffer A (25 mM Hepes pH 8, 200 mM NaCl, 2 mM β-mercaptoethanol and 5% glycerol). For co-crystallization of cA_6_-Cad1-CARF, 500 μM cA_6_, and for co-crystallization of cA_4_-Cad1-CARF, 1 mM cA_4_, were added to the 15 mg/ml of apo-Cad1-CARF (1–185) present in buffer A. For the screening of the crystallization condition hanging drop vapor diffusion method was used with 96 well hanging drop plates and the plates were set by the robot Mosquito (STP Labtech) with 1:1 protein to condition ratio. The plates were incubated at 20°C. Once the crystal hits were found, we shifted to 15 well hanging drop plates and set up drops by hand. The needle shaped crystals of apo-Cad1-CARF protein were grown in 0.1 M Tris-HCl pH 8.5, 2 M ammonium sulfate condition. cA_6_-Cad1-CARF complex formed small diamond shaped crystals in 0.1 M sodium acetate pH 4.5, 35% (v/v) MPD condition. cA_4_-Cad1-CARF complex formed large cubic crystals in 1 M lithium chloride, 0.1 M citric acid pH 5, 10% (w/v) PEG 6000. Single crystals were picked and frozen into liquid nitrogen after applying cryoprotectants formed by the respective crystallization conditions supplemented with 30% (v/v) glycerol.

#### X-ray diffraction data collection and structure determination

The x-ray diffraction data of the crystals of apo-Cad1-CARF, cA_6_-Cad1-CARF complex and cA_4_-Cad1-CARF complex were collected at the synchrotron beamline at Brookhaven National Lab (BNL). The datasets were collected at the oscillation range of 270° with oscillation width of 0.2°. The crystal-to-detector distance was kept at 200 mm for the data collection. For the cA_4_-Cad1-CARF complex crystals, the data were indexed, integrated, and scaled using HKL-2000 package^51^ and CCP4i2 software suite.^42^ In case of the crystals of apo-Cad1-CARF and cA_6_-Cad1-CARF complex the collected datasets were processed by BNL inbuilt automated processing tools and the integrated and scaled files were directly used for the molecular replacement using MOLREP program under CCP4i2 suit.^42^ For the molecular replacement Cad1-CARF model generated by AlphaFold2^52^ was used. The data collection and processing statistics are given in Table S1.

#### Cryo-EM sample preparation and imaging

The purified His_6_-tagged apo-Cad1 protein was concentrated to 17 μM using a 100 kDa cutoff Amicon Ultra Centrifugal Filter (Millipore Sigma) and used that for grid freezing. For Cad1-ATP bound sample 1 mM ATP was added to 17 μM apo-Cad1 protein and was incubated for an hour before grid freezing. The Cad1-cA_4_-ATP sample was prepared by mixing 400 μM cA_4_, 1 mM MgCl_2_ and 1 mM ATP to 20 μM apo-Cad1 protein and incubated for 1 h at 37°C temperature and 0.2 mM Fluorinated Octyl Maltoside detergent (FOM) was added to the reaction before grid freezing. The Cad1-cA_6_-ATP sample was prepared by addition of 200 μM cA_6_, 1 mM MgCl_2_ and 1 mM ATP to 20 μM apo-Cad1 protein and incubated overnight at 37°C. The reaction was loaded to a Superdex 200 10/300-increase column pre-equilibrated in buffer A (25 mM Hepes pH 8, 200 mM NaCl, 2 mM β-mercaptoethanol and 5% glycerol). The hexameric peak fraction was collected and concentrated to 17 μM and 0.2 mM Fluorinated Octyl Maltoside detergent (FOM) was added to the reaction prior to grid freezing.

We used UltrAuFoil R (1.2/1.3) grids (Quantifoil) for the sample preparation. The grids were glow-discharged for 1 min. The grids were frozen under 100% humidity, 12 s wait time, 2.5 s blot time and 0 blot force at 4°C temperature using a Vitrobot Mark IV (FEI). The data for apo-Cad1 and ATP-Cad1 were collected on a Titan Krios equipped with a K3 detector (Gatan) with an energy filter and with a slit width of 20–30 eV at NCCAT (National Center for Cryo-EM Access and Training). The data sets were collected with a pixel size of 0.532 Å at the super resolution mode (2x binning) and the images were recorded at a defocus range of −0.8 μm to −2.5 μm with a tilt series of 15°, 30° and 50°. The total electron dose was 53 electrons per Å^2^ with an exposure time of 2 s fractioned over 40 movie frames. The Cad1-cA_4_-ATP dataset was collected on Krios G4 with Falcon4i camera at MSKCC. The raw pixel size was 0.73 Å and the defocus range for the data collection was − 0.8 μm to −2.3 μm. The total electron dose was 28 electrons per Å^2^. The EER upsampling factor was 1 and EER number of fractions were 45. The Cad1-cA_6_-ATP dataset was collected on a Titan Krios equipped with a K3 detector (Gatan) with an energy filter and with a slit width of 20–30 eV without tilting the stage, at NCCAT (National Center for Cryo-EM Access and Training). The raw pixel size was 0.809Å and the defocus range was −0.8 μm to −2.4 μm. The total electron dose was 61 electrons per Å^2^ with an exposure time of 2 s fractioned over 50 movie frames. The data collection parameters are provided in Table S2.

#### Cryo-EM data processing and refinement

We used cryoSPARC v4.1.1 for the processing of apo-Cad1 and ATP-Cad1 datasets and cryoSPARC v4.4.1 was used for the processing of Cad1-cA_4_-ATP and Cad1-cA_6_-ATP/ITP datasets.^45^ The data processing and refinement statistics are provided in Table S2.

#### Processing of apo-Cad1

The movies were imported to cryoSPARC and subjected to patch motion correction. The motion corrected micrographs were used for the CTF (Contrast Transfer Function) estimation with patch CTF estimation job. 4694 micrographs were manually curated using manually curate exposures tool using a threshold of CTF fit resolution (Å) 2.6 Å −15 Å (Figures S9A–S9D). Blob picker with a diameter range of 150 Å to 200 Å was used to pick up 5,204,594 particles. Iterative rounds of 2D classification were performed with the extracted particles (box size 400 pixel) and 136,479 particles were used to build the ab-initio models and to train model using Topaz train tool. 7,386,739 particles were extracted, and 1,148,761 particles were selected using 2D classification. Hetero refinement was performed, and 451,854 particles were selected for further processing. The 3D classification job was performed with the selected particles, and they were classified into 4 classes. The best class was selected consisting of 110,836 particles and refined using non-uniform refinement with C3 symmetry to 3.6 Å following the standard FSC cutoff value of 0.143 (Figure S9D). The refined map was sharpened using volume sharpen tool using a B-factor of −100. The Alphafold2^52^ model of the CARF and the deaminase domains were fitted to the map using Chimera^44^ and the model was built using Coot.^47^ Phenix real-space refinement program was used to remove the outliers and refine the model with a model vs. data correlation value (CC mask) of 0.77.

#### Processing of ATP-bound apo-Cad1

4317 dose weighted micrographs were used for the data processing (Figures S9E–S9I). The parameters of the CTF were estimated using patch CTF estimation. Using blob picker 2,985,350 particles were picked and using 2D classification 294,209 particles were selected to build the ab initio models and for the Topaz model train program. Using Topaz trained model, we picked up 4,988,516 particles. By applying 2D classification we selected 761,583 particles for hetero refinement. The map could be refined to 3.96 Å by hetero refinement program with 419,796 particles. The refined map was further applied to homogeneous refinement and refined to 3.52 Å resolution. These particles were further classified into 4 classes by 3D classification. Two distinct classes were generated, one class with 106,838 particles and the other class having 100,094 particles. Each of the classes was further refined using homogeneous refinement to 3.74 Å for the first class and 3.68 Å for the second class. The first class was further refined using non-uniform refinement with C3 symmetry and the resolution improved to 3.38 Å following the standard FSC cutoff value of 0.143 (Figure S9G). The refined map was sharpened using the sharpen tool with −100 B-factor. The refined map of the second class was again refined using nonuniform refinement to 3.61 Å following the standard FSC cutoff value of 0.143 (Figure S9G) and sharpen tool was used to sharpen the map with −100 B factor. The AlphaFold2^52^ model of the CARF and the deaminase domains were fitted into the both the maps using Chimera^44^ and the model was built using Coot.^47^ The models were refined using Phenix real-space refinement program with a model vs. data correlation value of (CC mask) 0.81 and 0.80 for class1 and class2 respectively.

#### Processing of ATP-containing cA_4_-Cad1 complex

12,278 movies were imported to cryoSPARC and patch motion correction was performed (Figures S10A–S10D). Further, patch CTF estimation was carried out with the motion corrected micrographs. Two rounds of blob picking were performed with a diameter range of 190 Å to 300 Å, and 2,425,448 particles were picked up. Iterative rounds of 2D classification were performed and good 2D classes were selected as templates for template picker job and to build an ab-initio model. The template picker job picked up 2,609,801 particles. 2D classification jobs were performed to clean up the particles and we got a final set of 201,436 good particles with different views. Non-uniform refinement job was performed with those particles. The particles were then classified with 3D classification job into 3 classes with 74,964 particles in class 1; 65,733 particles in class 2; 60,739 particles in class 3. Out of the three classes, in class 1 and class 2, the CARF domains were broken. The class 3 was refined using non-uniform refinement to 3.2 Å resolution following the standard FSC cutoff value of 0.143 (Figure S10C). The AlphaFold2^52^ model of the CARF and the deaminase domain were fitted into the EM map using Chimera^44^ and the model was manually built in Coot.^47^ Phenix real space refinement was used for the final refinement of the model with a CC mask value of 0.86.

#### Processing of ATP-containing cA_6_-Cad1 complex

11,912 dose weighted micrographs were imported to cryoSPARC and patch CTF estimation job was performed (Figures S10E–S10H). The blob picker job was used to pick up 3,897,650 particles with a diameter range of 200 Å to 300 Å. Further, two rounds of 2D classification jobs were used to clean up the particles and 231,513 particles having different views were selected for Ab-initio model building. The same 2D classes were used as templates for the template picker job and 9,336,719 particles were extracted with a box size of 512 pixel (0.809 Å/pixel). Iterative rounds of 2D classification were used for particles clean up and duplicate particles were removed by remove duplicate job. Finally, 380,423 particles were used for non-uniform refinement job and followed by classified into 3 classes using 3D classification job. Out of the 3 classes, one of the CARF dimer domain was broken in class 3 but the other two classes looked similar, and the protein was intact in the EM maps. The particles from class 1 and class 2 were merged and final non-uniform refinement was performed with 271,802 particles and the map was refined to 3.6 Å resolution following the standard FSC cutoff value of 0.143 (Figure S10G). The Alphafold2^52^ models of the CARF and the deaminase domain were used for model fitting in the EM map using Chimera^44^ and the model was manually built in Coot^47^ and refined using Phenix real space refinement job with a CC mask value of 0.84.

### QUANTIFICATION AND STATISTICAL ANALYSIS

For cryo-EM structure determination, the numbers of particles used for each of the 3D reconstructions are listed (Figures S9 and S10), and fourier shell correlation (FSC) analyses for resolution determination were performed in cryoSPARC. All *p* values were calculated with GraphPad Prism 10.1.1 using a two-sided t test with Welch’s correction. Number and types of experimental replicates are described in figure legends.

## Supplementary Material

MMC6

MMC4

MMC7

MMC5

MMC2

MMC3

MMC1

1

## Figures and Tables

**Table:** KEY RESOURCES TABLE

REAGENT or RESOURCE	SOURCE	IDENTIFIER
Bacterial and virus strains		
Staphylococcus aureus RN4220	N/A	N/A
Escherichia coli Rosetta 2 (DE3) - Novagen	Millipore Sigma	71400–3
PhiNM1gamma6-GFP	Baca et al.^12^	N/A
Phi80alpha-vir	Banh et al.^29^	N/A
PhiNM4gamma4	Banh et al.^29^	N/A
PhiJ1	Banh et al.^29^	N/A
PhiJ2	Banh et al.^29^	N/A
PhiJ4	Banh et al.^29^	N/A
Chemicals, peptides, and recombinant proteins		
ATP	NEB	P0756S
dATP	NEB	N0440S
ITP	Sigma Aldrich	I0879–50MG
dITP	Thermo Fisher	R1191
Adenosine	Sigma Aldrich	A9251–1G
Inosine	Sigma Aldrich	I4125–1G
AMP	Sigma Aldrich	01930–5G
UTP	NEB	N0450L
CTP	NEB	N0450L
cA2	Biolog	C 088
cA3	Biolog	C 362
cA4	Biolog	C 335
cA6	Biolog	C 332
Deposited data		
Cad1 apo	This study	EMDB: 45241 and PDB: 9C67
Cad1 ATP asymmetric	This study	EMDB: 45245 and PDB: 9C6F
Cad1 ATP symmetric	This study	EMDB: 45244 and PDB: 9C6C
Cad1 cA4 ATP	This study	EMDB: 45277 and PDB: 9C77
Cad1 cA6 ATP	This study	EMDB: 45466 and PDB: 9CDB
Cad1 RNA-seq raw reads	This study	Deposited in NCBI Sequence Read Archive with BioProject: PRJNA1152273
apo-Cad1-CARF	This study	PDB: 9C6A
Cad1-CARF + cA6	This study	PDB: 9C68
Cad1-CARF-cA4	This study	PDB: 9C69
Oligonucleotides		
See Data S1	N/A	N/A
Recombinant DNA		
See Data S1	N/A	N/A
Software and algorithms		
CCP4i2 Suite	Winn et al.^42^	https://www.ccp4.ac.uk
Foldseek	van Kempen et al.^18^	https://search.foldseek.com/search
CRISPRCasTyper	Russel et al.^43^	https://crisprcastyper.crispr.dk/#/submit
UCSF Chimera	Pettersen et al.^44^	https://www.cgl.ucsf.edu/chimera/
cryoSPARC	Punjani et al.^45^	https://cryosparc.com
HISAT2	Kim et al.^46^	http://daehwankimlab.github.io/hisat2/
Coot	Emsley and Cowtan^47^	https://www2.mrc-lmb.cam.ac.uk/personal/pemsley/coot/
REDItools V2	Flati et al.^48^	https://github.com/BioinfoUNIBA/REDItools
Alphafold 3	Abramson et al.^49^	https://alphafoldserver.com
Python 3.9	Python	https://www.python.org/downloads/release/python-390/
Prism 10.1.1	GraphPad	https://www.graphpad.com
Prism 9.4.0	GraphPad	https://www.graphpad.com

## Highlights

- Type III CRISPR-Cas systems produce cyclic oligoadenylates upon phage recognition
- Type III-associated Cad1 hexamers are activated by cyclic oligoadenylate ligands
- Activated Cad1 catalyzes the deamination of ATP to ITP
- ITP accumulation is toxic to the bacterial host and prevents viral spread

## References

[1] Barrangou R, Fremaux C, Deveau H, Richards M, Boyaval P, Moineau S, Romero DA, and Horvath P (2007). CRISPR provides acquired resistance against viruses in prokaryotes. Science 315, 1709–1712. 10.1126/science.1138140. 17379808

[2] Marraffini LA, and Sontheimer EJ (2008). CRISPR interference limits horizontal gene transfer in staphylococci by targeting DNA. Science 322, 1843–1845. 10.1126/science.1165771. 19095942 PMC2695655

[3] Brouns SJJ, Jore MM, Lundgren M, Westra ER, Slijkhuis RJH, Snijders APL, Dickman MJ, Makarova KS, Koonin EV, and van der Oost J (2008). Small CRISPR RNAs guide antiviral defense in prokaryotes. Science 321, 960–964. 10.1126/science.1159689. 18703739 PMC5898235

[4] Carte J, Wang R, Li H, Terns RM, and Terns MP (2008). Cas6 is an endoribonuclease that generates guide RNAs for invader defense in prokaryotes. Genes Dev. 22, 3489–3496. 10.1101/gad.1742908. 19141480 PMC2607076

[5] Deltcheva E, Chylinski K, Sharma CM, Gonzales K, Chao Y, Pirzada ZA, Eckert MR, Vogel J, and Charpentier E (2011). CRISPR RNA maturation by trans-encoded small RNA and host factor RNase III. Nature 471, 602–607. 10.1038/nature09886. 21455174 PMC3070239

[6] Garneau JE, Dupuis MÈ, Villion M, Romero DA, Barrangou R, Boyaval P, Fremaux C, Horvath P, Magadán AH, and Moineau S (2010). The CRISPR/Cas bacterial immune system cleaves bacteriophage and plasmid DNA. Nature 468, 67–71. 10.1038/nature09523. 21048762

[7] Hale CR, Zhao P, Olson S, Duff MO, Graveley BR, Wells L, Terns RM, and Terns MP (2009). RNA-guided RNA cleavage by a CRISPR RNA-Cas protein complex. Cell 139, 945–956. 10.1016/j.cell.2009.07.040. 19945378 PMC2951265

[8] Jore MM, Lundgren M, van Duijn E, Bultema JB, Westra ER, Waghmare SP, Wiedenheft B, Pul U, Wurm R, Wagner R, (2011). Structural basis for CRISPR RNA-guided DNA recognition by Cascade. Nat. Struct. Mol. Biol 18, 529–536. 10.1038/nsmb.2019. 21460843

[9] Kazlauskiene M, Tamulaitis G, Kostiuk G, Venclovas Č, and Siksnys V (2016). Spatiotemporal Control of Type III-A CRISPR-Cas Immunity: Coupling DNA Degradation with the Target RNA Recognition. Mol. Cell 62, 295–306. 10.1016/j.molcel.2016.03.024. 27105119

[10] Kazlauskiene M, Kostiuk G, Venclovas Č, Tamulaitis G, and Siksnys V (2017). A cyclic oligonucleotide signaling pathway in type III CRISPR-Cas systems. Science 357, 605–609. 10.1126/science.aao0100. 28663439

[11] Niewoehner O, Garcia-Doval C, Rostøl JT, Berk C, Schwede F, Bigler L, Hall J, Marraffini LA, and Jinek M (2017). Type III CRISPR-Cas systems produce cyclic oligoadenylate second messengers. Nature 548, 543–548. 10.1038/nature23467. 28722012

[12] Baca CF, Yu Y, Rostøl JT, Majumder P, Patel DJ, and Marraffini LA (2024). The CRISPR effector Cam1 mediates membrane depolarization for phage defence. Nature 625, 797–804. 10.1038/s41586-023-06902-y. 38200316 PMC10808066

[13] Rostøl JT, Xie W, Kuryavyi V, Maguin P, Kao K, Froom R, Patel DJ, and Marraffini LA (2021). The Card1 nuclease provides defence during type III CRISPR immunity. Nature 590, 624–629. 10.1038/s41586-021-03206-x. 33461211 PMC7906951

[14] Jiang W, Samai P, and Marraffini LA (2016). Degradation of phage transcripts by CRISPR-associated RNases enables type III CRISPR-Cas immunity. Cell 164, 710–721. 10.1016/j.cell.2015.12.053. 26853474 PMC4752873

[15] McMahon SA, Zhu W, Graham S, Rambo R, White MF, and Gloster TM (2020). Structure and mechanism of a Type III CRISPR defence DNA nuclease activated by cyclic oligoadenylate. Nat. Commun 11, 500. 10.1038/s41467-019-14222-x. 31980625 PMC6981274

[16] Niewoehner O, and Jinek M (2016). Structural basis for the endoribonuclease activity of the type III-A CRISPR-associated protein Csm6. RNA 22, 318–329. 10.1261/rna.054098.115. 26763118 PMC4748810

[17] Zhu W, McQuarrie S, Grüschow S, McMahon SA, Graham S, Gloster TM, and White MF (2021). The CRISPR ancillary effector Can2 is a dual-specificity nuclease potentiating type III CRISPR defence. Nucleic Acids Res. 49, 2777–2789. 10.1093/nar/gkab073. 33590098 PMC7969007

[18] van Kempen M, Kim SS, Tumescheit C, Mirdita M, Lee J, Gilchrist CLM, Söding J, and Steinegger M (2024). Fast and accurate protein structure search with Foldseek. Nat. Biotechnol 42, 243–246. 10.1038/s41587-023-01773-0. 37156916 PMC10869269

[19] Makarova KS, Anantharaman V, Grishin NV, Koonin EV, and Aravind L (2014). CARF and WYL domains: ligand-binding regulators of prokaryotic defense systems. Front. Genet 5, 102. 10.3389/fgene.2014.00102. 24817877 PMC4012209

[20] Makarova KS, Timinskas A, Wolf YI, Gussow AB, Siksnys V, Venclovas Č, and Koonin EV (2020). Evolutionary and functional classification of the CARF domain superfamily, key sensors in prokaryotic antivirus defense. Nucleic Acids Res. 48, 8828–8847. 10.1093/nar/gkaa635. 32735657 PMC7498327

[21] Horinouchi S, and Weisblum B (1982). Nucleotide sequence and functional map of pC194, a plasmid that specifies inducible chloramphenicol resistance. J. Bacteriol 150, 815–825. 10.1128/jb.150.2.815-825.1982. 6950931 PMC216434

[22] Rostøl JT, and Marraffini LA (2019). Non-specific degradation of transcripts promotes plasmid clearance during type III-A CRISPR-Cas immunity. Nat. Microbiol 4, 656–662. 10.1038/s41564-018-0353-x. 30692669 PMC6430669

[23] Kreiswirth BN, Löfdahl S, Betley MJ, O’Reilly M, Schlievert PM, Bergdoll MS, and Novick RP (1983). The toxic shock syndrome exotoxin structural gene is not detectably transmitted by a prophage. Nature 305, 709–712. 10.1038/305709a0. 6226876

[24] Jia N, Jones R, Yang G, Ouerfelli O, and Patel DJ (2019). CRISPR-Cas III-A Csm6 CARF Domain Is a Ring Nuclease Triggering Stepwise cA4 Cleavage with ApA>p Formation Terminating RNase Activity. Mol. Cell 75, 944–956.e6. 10.1016/j.molcel.2019.06.014. 31326273 PMC6731128

[25] Larson ET, Deng W, Krumm BE, Napuli A, Mueller N, Van Voorhis WC, Buckner FS, Fan E, Lauricella A, DeTitta G, (2008). Structures of substrate- and inhibitor-bound adenosine deaminase from a human malaria parasite show a dramatic conformational change and shed light on drug selectivity. J. Mol. Biol 381, 975–988. 10.1016/j.jmb.2008.06.048. 18602399 PMC2600493

[26] Wilson DK, Rudolph FB, and Quiocho FA (1991). Atomic structure of adenosine deaminase complexed with a transition-state analog: understanding catalysis and immunodeficiency mutations. Science 252, 1278–1284. 10.1126/science.1925539. 1925539

[27] Gao L, Altae-Tran H, Böhning F, Makarova KS, Segel M, Schmid-Burgk JL, Koob J, Wolf YI, Koonin EV, and Zhang F (2020). Diverse enzymatic activities mediate antiviral immunity in prokaryotes. Science 369, 1077–1084. 10.1126/science.aba0372. 32855333 PMC7985843

[28] Goldberg GW, Jiang W, Bikard D, and Marraffini LA (2014). Conditional tolerance of temperate phages via transcription-dependent CRISPR-Cas targeting. Nature 514, 633–637. 10.1038/nature13637. 25174707 PMC4214910

[29] Banh DV, Roberts CG, Morales-Amador A, Berryhill BA, Chaudhry W, Levin BR, Brady SF, and Marraffini LA (2023). Bacterial cGAS senses a viral RNA to initiate immunity. Nature 623, 1001–1008. 10.1038/s41586-023-06743-9. 37968393 PMC10686824

[30] Mogila I, Tamulaitiene G, Keda K, Timinskas A, Ruksenaite A, Sasnauskas G, Venclovas Č, Siksnys V, and Tamulaitis G (2023). Ribosomal stalk-captured CARF-RelE ribonuclease inhibits translation following CRISPR signaling. Science 382, 1036–1041. 10.1126/science.adj2107. 38033086

[31] Molina R, Stella S, Feng M, Sofos N, Jauniskis V, Pozdnyakova I, López-Méndez B, She Q, and Montoya G (2019). Structure of Csx1-cOA4 complex reveals the basis of RNA decay in Type III-B CRISPR-Cas. Nat. Commun 10, 4302. 10.1038/s41467-019-12244-z. 31541109 PMC6754442

[32] Vidal AE, Yagüe-Capilla M, Martínez-Arribas B, García-Caballero D, Ruiz-Pérez LM, and González-Pacanowska D (2022). Inosine triphosphate pyrophosphatase from Trypanosoma brucei cleanses cytosolic pools from deaminated nucleotides. Sci. Rep 12, 6408. 10.1038/s41598-022-10149-4. 35436992 PMC9016069

[33] Abolhassani N, Iyama T, Tsuchimoto D, Sakumi K, Ohno M, Behmanesh M, and Nakabeppu Y (2010). NUDT16 and ITPA play a dual protective role in maintaining chromosome stability and cell growth by eliminating dIDP/IDP and dITP/ITP from nucleotide pools in mammals. Nucleic Acids Res. 38, 2891–2903. 10.1093/nar/gkp1250. 20081199 PMC2875033

[34] Menezes MR, Waisertreiger ISR, Lopez-Bertoni H, Luo X, and Pavlov YI (2012). Pivotal role of inosine triphosphate pyrophosphatase in maintaining genome stability and the prevention of apoptosis in human cells. PLoS One 7, e32313. 10.1371/journal.pone.0032313. 22384212 PMC3288088

[35] Zheng J, Singh VK, and Jia Z (2005). Identification of an ITPase/XTPase in Escherichia coli by structural and biochemical analysis. Structure 13, 1511–1520. 10.1016/j.str.2005.07.007. 16216582

[36] Behmanesh M, Sakumi K, Abolhassani N, Toyokuni S, Oka S, Ohnishi YN, Tsuchimoto D, and Nakabeppu Y (2009). ITPase-deficient mice show growth retardation and die before weaning. Cell Death Differ. 16, 1315–1322. 10.1038/cdd.2009.53. 19498443

[37] Duncan-Lowey B, Tal N, Johnson AG, Rawson S, Mayer ML, Doron S, Millman A, Melamed S, Fedorenko T, Kacen A, (2023). Cryo-EM structure of the RADAR supramolecular anti-phage defense complex. Cell 186, 987–998.e15. 10.1016/j.cell.2023.01.012. 36764290 PMC9994260

[38] Gao Y, Luo X, Li P, Li Z, Ye F, Liu S, and Gao P (2023). Molecular basis of RADAR anti-phage supramolecular assemblies. Cell 186, 999–1012.e20. 10.1016/j.cell.2023.01.026. 36764292

[39] Hsueh BY, Severin GB, Elg CA, Waldron EJ, Kant A, Wessel AJ, Dover JA, Rhoades CR, Ridenhour BJ, Parent KN, (2022). Phage defence by deaminase-mediated depletion of deoxynucleotides in bacteria. Nat. Microbiol 7, 1210–1220. 10.1038/s41564-022-01162-4. 35817890 PMC9830645

[40] Tal N, Millman A, Stokar-Avihail A, Fedorenko T, Leavitt A, Melamed S, Yirmiya E, Avraham C, Brandis A, Mehlman T, (2022). Bacteria deplete deoxynucleotides to defend against bacteriophage infection. Nat. Microbiol 7, 1200–1209. 10.1038/s41564-022-01158-0. 35817891

[41] Wilson DK, and Quiocho FA (1993). A pre-transition-state mimic of an enzyme: X-ray structure of adenosine deaminase with bound 1-deazaadenosine and zinc-activated water. Biochemistry 32, 1689–1694. 10.1021/bi00058a001. 8439534

[42] Winn MD, Ballard CC, Cowtan KD, Dodson EJ, Emsley P, Evans PR, Keegan RM, Krissinel EB, Leslie AGW, McCoy A, (2011). Overview of the CCP4 suite and current developments. Acta Crystallogr. D Biol. Crystallogr 67, 235–242. 10.1107/S0907444910045749. 21460441 PMC3069738

[43] Russel J, Pinilla-Redondo R, Mayo-Muñoz D, Shah SA, and Sørensen SJ (2020). CRISPRCasTyper: Automated Identification, Annotation, and Classification of CRISPR-Cas Loci. CRISPR J. 3, 462–469. 10.1089/crispr.2020.0059. 33275853

[44] Pettersen EF, Goddard TD, Huang CC, Couch GS, Greenblatt DM, Meng EC, and Ferrin TE (2004). UCSF Chimera–a visualization system for exploratory research and analysis. J. Comput. Chem 25, 1605–1612. 10.1002/jcc.20084. 15264254

[45] Punjani A, Rubinstein JL, Fleet DJ, and Brubaker MA (2017). cryoSPARC: algorithms for rapid unsupervised cryo-EM structure determination. Nat. Methods 14, 290–296. 10.1038/nmeth.4169. 28165473

[46] Kim D, Paggi JM, Park C, Bennett C, and Salzberg SL (2019). Graph-based genome alignment and genotyping with HISAT2 and HISAT-genotype. Nat. Biotechnol 37, 907–915. 10.1038/s41587-019-0201-4. 31375807 PMC7605509

[47] Emsley P, and Cowtan K (2004). Coot: model-building tools for molecular graphics. Acta Crystallogr. D Biol. Crystallogr 60, 2126–2132. 10.1107/S0907444904019158. 15572765

[48] Flati T, Gioiosa S, Spallanzani N, Tagliaferri I, Diroma MA, Pesole G, Chillemi G, Picardi E, and Castrignanò T (2020). HPC-REDItools: a novel HPC-aware tool for improved large scale RNA-editing analysis. BMC Bioinformatics 21, 353. 10.1186/s12859-020-03562-x. 32838738 PMC7446188

[49] Abramson J, Adler J, Dunger J, Evans R, Green T, Pritzel A, Ronneberger O, Willmore L, Ballard AJ, Bambrick J, (2024). Accurate structure prediction of biomolecular interactions with AlphaFold 3. Nature 630, 493–500. 10.1038/s41586-024-07487-w. 38718835 PMC11168924

[50] Madeira F, Park YM, Lee J, Buso N, Gur T, Madhusoodanan N, Basutkar P, Tivey ARN, Potter SC, Finn RD, and Lopez R (2019). The EMBL-EBI search and sequence analysis tools APIs in 2019. Nucleic Acids Res. 47, W636–W641. 10.1093/nar/gkz268. 30976793 PMC6602479

[51] Otwinowski Z, and Minor W (1997). Processing of X-ray diffraction data collected in oscillation mode. Methods Enzymol. 276, 307–326. 10.1016/S0076-6879(97)76066-X. 27754618

[52] Varadi M, Anyango S, Deshpande M, Nair S, Natassia C, Yordanova G, Yuan D, Stroe O, Wood G, Laydon A, (2022). AlphaFold Protein Structure Database: massively expanding the structural coverage of protein-sequence space with high-accuracy models. Nucleic Acids Res. 50, D439–D444. 10.1093/nar/gkab1061. 34791371 PMC8728224

