# Supplementary material for: The CRISPR-associated adenosine deaminase Cad1 converts ATP to ITP to provide antiviral immunity
Source: Cell. Author manuscript; Available in PMC 2024 Dec 14. (PMC11645235; doi:10.1016/j.cell.2024.10.002)
Supplement: MMC1 [file NIHMS2028211-supplement-MMC1.pdf]

Cell, Volume *187*

Supplemental information

The CRISPR-associated adenosine deaminase Cad1 converts ATP to ITP to provide antiviral immunity

Christian F. Baca, Puja Majumder, James H. Hickling, Linzhi Ye, Marianna Teplova, Sean F. Brady, Dinshaw J. Patel, and Luciano A. Marraffini

Table S1. X-ray data collection and refinement statistics, related to STAR Methods

	apo Cad1-CARF (PDB: 9C6A)	Cad1-CARF + cA_6 (PDB: 9C68)	Cad1-CARF + cA_4 (PDB: 9C69)
**Data Collection**			
Wavelength	0.9793	0.9793	0.9793
Space group	$P2_12_12_1$	$P6_222$	I432
**Cell dimension**			
a, b, c (Å)	53.615, 96.801, 155.722	85.925, 85.925, 188.721	189.308, 189.308, 189.308
α, β, γ (º)	90, 90, 90	90, 90, 120	90, 90, 90
Resolution range (Å)	77.8 -3.6 (3.7-3.6)	41.9 – 1.8 (1.9 -1.8)	47.33 – 2.4 (2.5 - 2.4)
R_{sym} or R_{merge}	0.1838 (0.6441)	0.02715 (1.372)	0.099 (0.537)
Mean $I / \sigma I$	2.15 (0.98)	10.04 (0.53)	42.12 (12.45)
Completeness (%)	99.9 (100)	99.95 (99.86)	98.86 (100)
Redundancy	2.0 (2.0)	2.0 (2.0)	43.1 (43.0)
CC1/2	0.966 (0.558)	0.999 (0.532)	1 (0.980)
**Refinement**			
Resolution (Å)	3.6	1.8	2.4
No. of reflection	9970 (955)	37687 (3670)	22654 (2238)
R_{work}/ R_{free}	25.5/31.5	26.7/31.2	20.15 (25.2)
No. of non-hydrogen atoms	5332	2819	2782
Ramachandran favored (%)	94.51	96.59	97.56
Ramachandran allowed (%)	5.49	2.48	2.44
Ramachandran outliers (%)	0.00	0.93	0.00
Rotamer outliers (%)	0.00	3.48	2.76
Clashscore	5.61	11.9	4.64
Average *B*-factor	62.14	43.63	37.77
R.M.S. deviations			
Bond lengths (Å)	0.003	0.010	0.009
Bond angles (º)	0.66	1.42	1.07

Table S2. Cryo-EM data collection and refinement statistics, related to STAR Methods

Parameters	Apo-Cad1 (PDB: 9C67 / EMD-45241)	ATP-Cad1 Symmetric (PDB: 9C6C / EMD-45244)	ATP-Cad1 Asymmetric (PDB: 9C6F / EMD-45245)	cA_4-Cad1-ATP (PDB: 9C77 / EMD-45277)	cA_6-Cad1-ATP (PDB: 9CDB / EMD-45466)
**Data Collection/processing**					
Microscope	Titan GIF/K3	Titan GIF/K3	Titan GIF/K3	Krios G4	Titan GIF/K3
Voltage (kV)	300	300	300	300	300
Detector	Gatan K3	Gatan K3	Gatan K3	Falcon 4i	Gatan K3
Magnification	81000	81000	81000	165000	105000
Defocus range (µm)	-0.8 to -2.5	-0.8 to -2.5	-0.8 to -2.5	-0.8 to -2.3	-0.8 to -2.4
Pixel size (Å/px)	0.53	0.53	0.53	0.73	0.809
Electron exposure (e^-/Å^2)	53	53	53	28	61
Exposure time (s)	2	2	2	2.9	2
Tilt series (°)	15, 30, 50	15, 30, 50	15, 30, 50	-	-
Energy Filter (eV slit)	20	20	20	10	20
Symmetry imposed	C3	C3	C1	C1	C1
EER upsampling factor	-	-	-	1	-
EER number of fractions	-	-	-	45	-
Initial particle number	7,386,739	4,988,516	4,988,516	2,609,801	9,336,719
Final particle number	110,836	106,838	100,094	60,739	271,802
Map Resolution (Å)	3.6	3.4	3.6	3.2	3.6
FSC threshold	0.143	0.143	0.143	0.143	0.143
**Refinement**					
Initial model	AlphFold2 model	AlphFold2 model	AlphFold2 model	AlphFold2 model	AlphFold2 model
Map resolution (masked) (FSC 0.143)	3.6	3.4	3.4	3.2	3.6
**Model Composition**					
Non-Hydrogen atoms	26783	27808	28356	28792	28605
Protein residues	3392	3516	3588	3575	3576
Ligands	MG: 6	MG: 6, ATP: 3	MG: 6, ATP: 3	MG:6, ATP:12	MG:6, ATP:7 LIG:5
**RMS deviations**					
Bond lengths	0.003	0.003	0.003	0.003	0.003
Bond angles	0.771	0.665	0.697	0.607	0.576
**Validation**					
Refined model CC (box)	0.9	0.82	0.82	0.84	0.87
Molprobity score	2.1	2.01	2.11	2.01	2.01
**Ramachandran plot (%)**					
Favored	94.82	94.47	93.91	94.57	94.18
Allowed	4.88	5.24	5.81	5.23	5.65
Disallowed	0.3	0.29	0.28	0.20	0.17
