# Supplementary material for: The CRISPR-associated adenosine deaminase Cad1 converts ATP to ITP to provide antiviral immunity
Source: Cell. Author manuscript; Available in PMC 2024 Dec 14. (PMC11645235; doi:10.1016/j.cell.2024.10.002)
Supplement: 1 [file NIHMS2028211-supplement-1.pdf]

CellPress
OPEN ACCESS

Supplemental figures

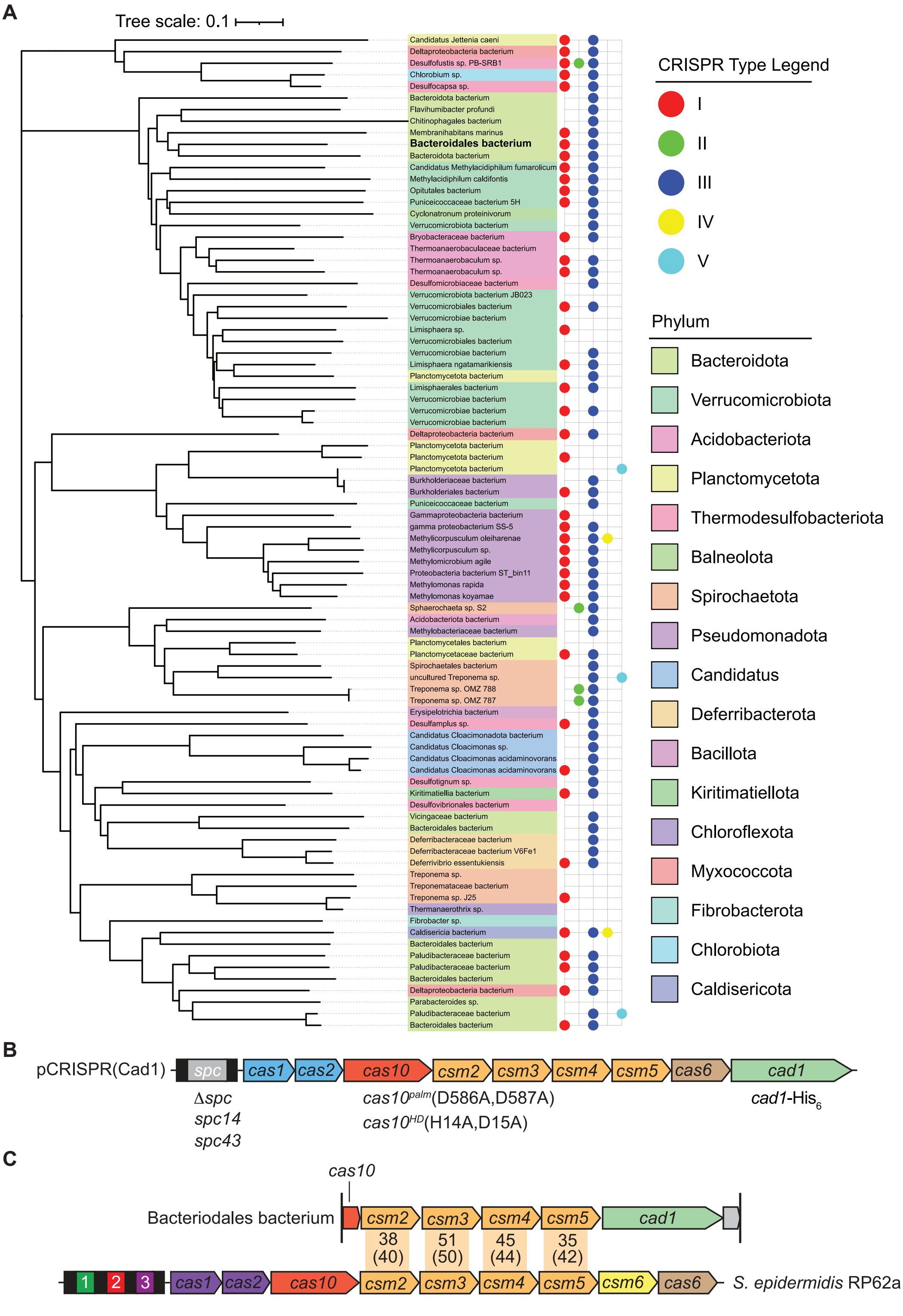

(legend on next page)

Figure S1. Cad1 homologs associate with type III CRISPR-Cas systems, related to Figure 1

(A) Phylogenetic tree of Cad1 homologs indicating the organism of origin; different phyla are highlighted with different colors. Also shown are the CRISPR types found within the genome of the host bacterium, determined using CRISPRCasTyper.[52]

(B) Schematic of the *Staphylococcus epidermidis* RP62 type III-A CRISPR-Cas locus used in this study, indicating the different spacers and amino acid substitutions tested. *cad1* or *cad1-His$_6$* were inserted at the end of the locus.

(C) Comparison of the type III-A systems of the unknown *Bacteriodales* bacterium where Cad1 was found and *Staphylococcus epidermidis* RP62. Black boxes, CRISPR repeats; colored and numbered boxes, CRISPR spacers. Numbers indicate the % homology at the amino acid sequence level. Numbers in parenthesis indicate the % homology at the DNA sequence level.

A

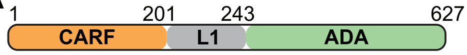

B

```
Bacteriodales       MSRVLLCSAGHSSMVVPEAFHAVPE--GFEEVHVFTTDSEKFNPVVLNDFFHSLPNVRFSITKCHGLADILNERDFEFYQEMLWQWY
Desulfomicrobiaceae MAQ-LFITVGTAPAVVLEAVGLMLQHQRLDAVHVIASASKRIETTMLHQFFQAQ-KMDYSITRVADFSDLGTPNDHERFEEVLLRWL
                    *:: *: :.* :. ** **.  : :   :: ***::: *:::::.:*::**::  :: :***:  .::*: . .*.* ::*:* :*

Bacteriodales       LTKMPDN--ELPYVCLSGGIKSMSASLQKAATLFGAQSVFHVLA----------DNNPRNIEEMFDALQKGQIHFIEMGYEPGWAAL
Desulfomicrobiaceae LHRMHPLPRTERWVCLAGGFKTMSAAMQQAAELFGAQEVFHVLATPMFGAEAGRKREPSTAEEVEEALRRHGVHWICLGGQTGWPQV
                    * :*         :***:**:*:***::*:** *****.******           ..:* . **: :**::  :*:* :* :.**. :

Bacteriodales       RRLKKIL---PINEGCSRDNFKPL--ISKSIEEILSNVKIM---ASD-TGKSNQLPFPSLAILPPIAQQWLQLPLSA-NDGAWIQNL
Desulfomicrobiaceae QRLRSADYPLVLEKGDAVGEFKAYPTPGSSLREYVAAMRRRSIHVSHHFERLARLPFPILASWSPQELQWLEEPLDGENDRTWVRSL
                    :**:.       :::* : .:**.    ..*:.* :: ::     .*.   :  :**** **   .*   ***: **.. ** :*::.*

Bacteriodales       PKVDLHCHLGGFATSGSLLDQVRGAASEPDLIDRTFSPQEIAGWPRSHKSISLDKYMELGNANGSKLLKDKGCLIRQVELLYQSLVN
Desulfomicrobiaceae PKIDLHCHLGGFATHGTDLEAVRQAACFPEKLPTQPVPEFPPGWPVPPEPITLAEYTRLGDATGTRLLRDPGCLQRHCQLLYETLAE
                    **:*********** *: *: ** **. *: :      *:   .***  . :.*:* :*  .**:*.*::**:* *** *: :***::*.:

Bacteriodales       DNVAYAEIRCSPNNYADKNKNRSAWVVLQDINDTFTRLITEAKQKNQFYCHVNLLVIASRKFSGDL-SDISKHLALAITAMQQGEGV
Desulfomicrobiaceae DGIVYAEVRCSPANYAT--DERSPWEVLTDIRNIFQAC-----MQDD-RCHVNLILIATRRQSGNFRTHMARHISLAVTAAERWQEG
                    *.:.***:**** ***    .:**.* ** **.: *          :::  *****::**:*: **:: :.:::*::**:** :: :

Bacteriodales       DNVAYAEIRCSPNNYADKNKNRSAWVVLQDINDTFTRLITEAKQKNQFYCHVNLLVIASRKFSGDL-SDISKHLALAITAMQQGEGV
Desulfomicrobiaceae DGIVYAEVRCSPANYAT--DERSPWEVLTDIRNIFQAC-----MQDD-RCHVNLILIATRRQSGNFRTHMARHISLAVTAAERWQEG
                    *.:.***:**** ***    .:**.* ** **.: *          :::  *****::**:*: **:: :.:::*::**:** :: :

Bacteriodales       PYANYQIKGFAPM--PN---FSAL---------YPLKKYLEAGILVSVNTDNIGISGANLSENLLILADLCPGISRMDVLTIIRNSI
Desulfomicrobiaceae PYANYQIRHPAPNLVPQQECFACLDVPATDRMTYPLREYLRKGVAVTINTDNIGISQANLTDNWIFTARLCPGITRMELLATLRHSL
                    *******:  **    *:   *:.*         ***::**. *: *::******** ***::* :: * *****:**::*: :*:*:

Bacteriodales       ETAFISHDFRMELLKFFDRKIYDVCLISIKN
Desulfomicrobiaceae EAAFIGHNERQGLLEKISHHLNPQWS---TP
                    *:***.*: *  **: :.:::       .
```

C D E F G H

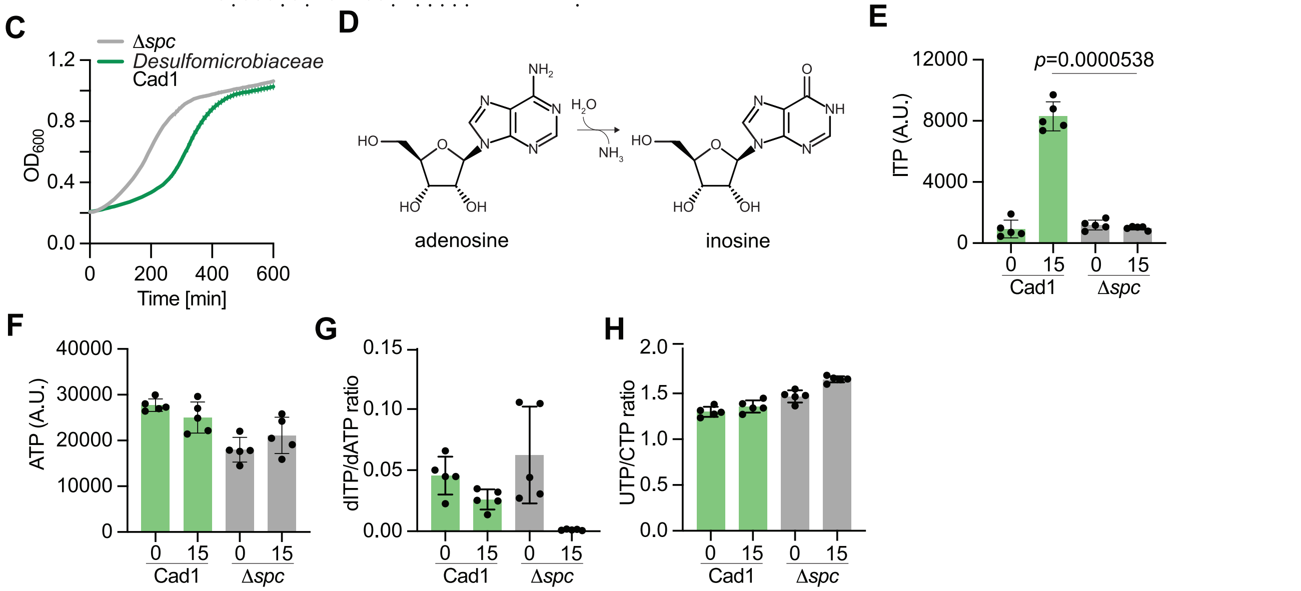

Figure S2. *In vivo* ATP to ITP conversion, related to Figure 1

(A) Domain architecture of *Desulfomicrobiaceae* Cad1. The protein contains a N-terminal CARF domain followed by a linker (L1) and a C-terminal adenosine deaminase (ADA) domain. Numbers indicate amino acid lengths.

(B) T-COFFEE alignment of *Bacteriodales* and *Desulfomicrobiaceae* Cad1 proteins. (*) indicates identical residues; (:) indicates strongly similar residues; (.) indicates weakly similar residues. The proteins are 40% identical and share 60% of similarity.

(C) Growth of staphylococci carrying pTarget and pCRISPR with (teal curve) or without (gray curve) a targeting spacer, measured as OD_{600} after the addition of aTc. Mean of three biological triplicates, ±SEM, is reported.

(D) Adenosine deamination reaction. Cad1 is predicted to convert the C6 amine group of adenosine to a carbonyl group, generating inosine.

(E) Raw values of ITP for each of the samples used to calculate ITP/ATP ratios in Figure 1G.

(legend continued on next page)

(F) Same as (E) but for ATP values.
(G) Quantification of dITP/dATP ratios from bacterial lysates. Staphylococci harboring pTarget and pCRISPR(Δspc) or pCRISPR(Cad1) were either collected before (0 min) or after (15 min) incubation with aTc. Cells were pelleted, and metabolites were extracted and analyzed via LC-MS measurements. Peaks for the masses of ATP and ITP were integrated to produce a single value proportional with the concentration of the molecule in the sample. These values were used to calculate the ITP/ATP ratio. Mean of five biological replicates ±SEM, is reported. *p* values, obtained with a two-sided t test with Welch's correction, are shown.
(H) Same as (G) but for UTP/CTP ratios.

CellPress
OPEN ACCESS

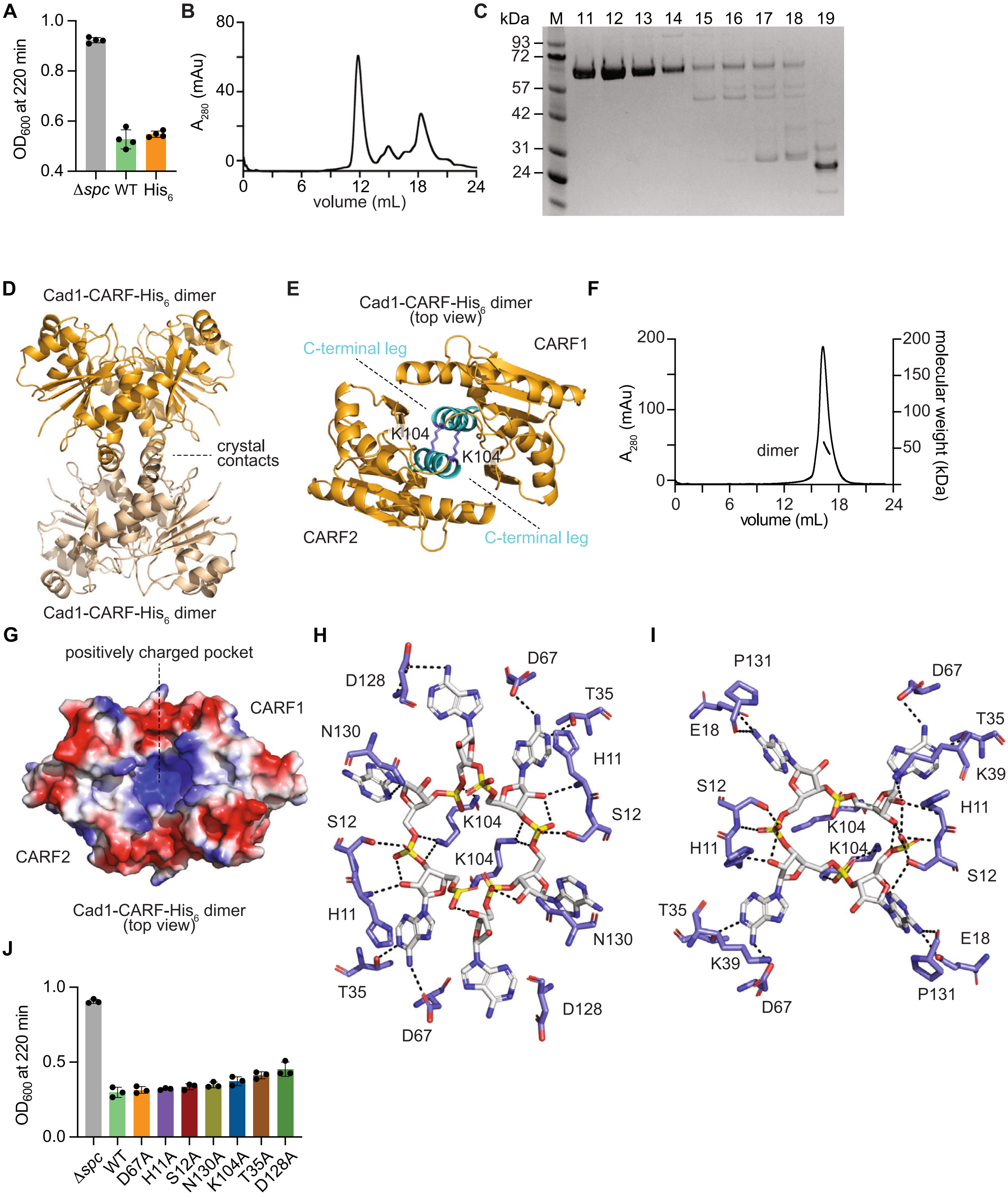

Figure S3. Properties of the Cad1-CARF-His$_6$, related to Figure 2

(A) Growth of staphylococci carrying pTarget and different pCRISPR constructs, measured as the OD_{600} value after 220 min of addition of aTc. Mean of four biological triplicates, ±SEM, is reported. Results show that both wild-type Cad1 ("WT") as well as a version harboring a C-terminal hexahistidyl tag extension ("His$_6$") are equally capable of inducing staphylococcal arrest.

(B) Size-exclusion chromatogram of Cad1-His$_6$ co-purified with bound cOAs from staphylococcal cells in which the type III-A CRISPR-Cas response was induced with aTc.

(C) SDS-PAGE of the different fractions (11–19) from (B). Fractions 11–13 were used for the identification of the co-purified cOAs shown in Figure 2A. The kDa value of molecular weight markers (M) is shown.

(D) The asymmetric unit of the crystal structure of apo-Cad1-CARF-His$_6$ showing crystal-packing contacts between dimers.

(legend continued on next page)

(E) Top view of the apo-Cad1-CARF-His_6 dimer illustrating that CARF1 and CARF2 monomers are arranged in a pseudo-2-fold symmetry. The C-terminal legs from each monomer are colored in cyan. K104 residues from both CARF1 and CARF2 are shown in stick representation.
(F) SECMALS profile of Cad1-CARF-His_6 displayed a molecular weight of 50 kDa (±0.715%) in solution, corresponding to a dimer.
(G) Top view of the apo-Cad1-CARF-His_6 dimer showing the surface electrostatic potential. The positively charged pocket at the dimeric interface is indicated.
(H and I) Amino acid residues of the Cad1-CARF-His_6 dimer protein involved in interactions with cA_6 (H) and cA_4 (I), with polar interactions indicated by black dashed lines.
(J) Growth of staphylococci carrying pTarget and different pCRISPR(Cad1) plasmids harboring alanine substitutions of amino acid residues that interact with cA_6 (shown in H) and cA_4 (shown in I). Growth was measured as the OD_{600} value after 220 min of addition of aTc. Mean of three biological triplicates, ±SEM, is reported.

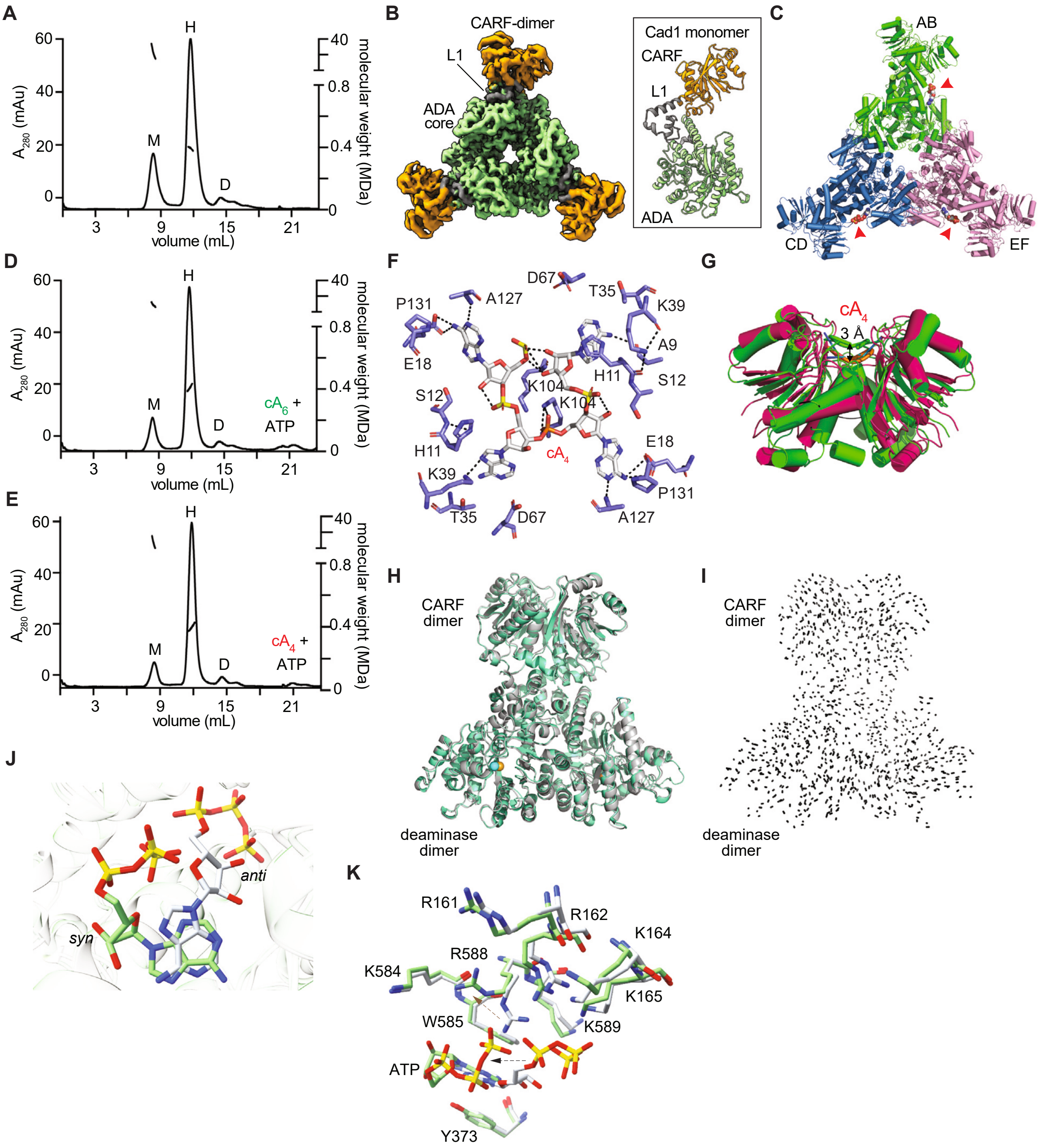

Figure S4. Characterization of hexameric Cad1 complexes, related to Figure 3

(A) SECMALS analysis of apo-Cad1 after a 14-h incubation at room temperature, showing the formation of a hexamer (H), a dimer (D), and a megadalton species (M).

(B) Cryo-EM structure of a trimer of Cad1 dimers at 3.6 Å resolution displaying the dimeric CARF head domains (orange), the L1 linker (gray), and dimeric adenosine deaminase (ADA) domains (green). A Cad1 monomer is shown in the inset.

(C) The cryo-EM structure of ATP-bound Cad1 hexamer displaying the AB, CD, and EF dimeric units in green, blue, and pink cylindrical representation, respectively. As opposed to the structure shown in Figure 3B, the three ATP molecules are bound asymmetrically to the inter-domain sites.

(D) Same as (A) but for Cad1 after incubation with cA_6 and ATP.

(E) Same as (A) but for Cad1 after incubation with cA_4 and ATP.

(F) Amino acid residues of the Cad1 hexamer involved in interactions with cA_4. Black dotted lines show hydrogen bond interactions.

(legend continued on next page)

(G) Comparison of cA_4 binding to Cad1-CARF-His_6 (red) and to the CARF domain of the full-length cA4-Cad1-ATP hexameric structure (green). The cA_4 molecule has shifted 3 Å in the upward direction in the cryo-EM structure, shown by a double-headed black arrow. In addition, in the cryo-EM structure, the angle between the C-terminal legs has decreased to 57°. The RMSD between the CARF domains of the crystal and cryo-EM structures is 2.8 Å.

(H) Superposition of one of the dimers from the apo-Cad1-ATP hexameric cryo-EM structure (silver) to the one of the dimers from the hexameric cA_4-Cad1-ATP structure (green); RMSD is 0.63 Å. ATP/cA_4 are not shown. The metals at the deaminase pocket of apo-Cad1 and cA_4 bound Cad1 are shown as cyan and orange spheres, respectively.

(I) Atom displacements for the structures shown in (H) are shown with black vectors.

(J) Superposition of ATP molecules bound to the ATP binding pocket of apo-Cad1 (silver backbone, *syn*-conformation) and cA_4-bound Cad1 (green backbone, *anti*-conformation).

(K) Same as (J) but also showing the relevant residues of the inter-domain ATP binding pocket. The black dotted arrow shows the displacement of the phosphate groups of the ATP molecule in the cA_4-bound Cad1 structure with respect to the apo structure. The brown dotted arrow indicates the flip of the side chain of R588 that occurs upon cA_4 binding.

CellPress
OPEN ACCESS

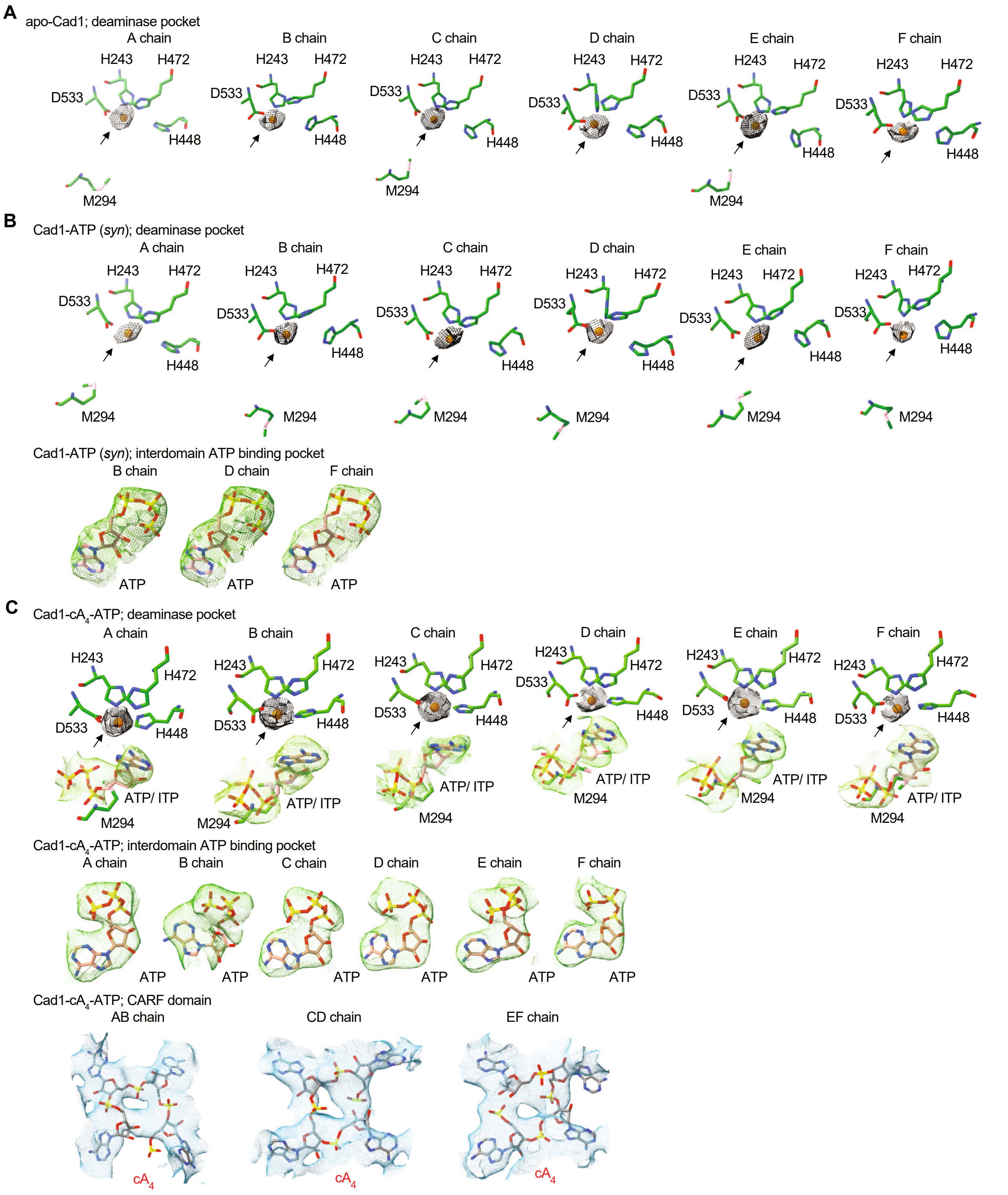

(legend on next page)

Figure S5. The metal and ligand Coulomb potential in Cad1 cryo-EM maps, related to Figure 3

(A) The Coulomb potential at each of the deaminase pocket of apo-Cad1 cryo-EM map corresponding to the modeled magnesium ion is displayed in black mesh representation.

(B) The Coulomb potential corresponding to the modeled magnesium ions (black mesh) at each of the deaminase active site and ATP molecule at the inter-domain ATP binding pockets (green mesh) in the ATP-Cad1 (symmetric) cryo-EM map are presented.

(C) The density corresponding the modeled magnesium ion (black mesh) and ATP/ITP (green mesh) at the deaminase pocket, ATP (green mesh) at the inter-domain ATP binding pocket, and cA_4 (blue mesh) bound to the dimeric CARF domain in the ATP-Cad1-cA_4 cryo-EM map are displayed.

CellPress
OPEN ACCESS

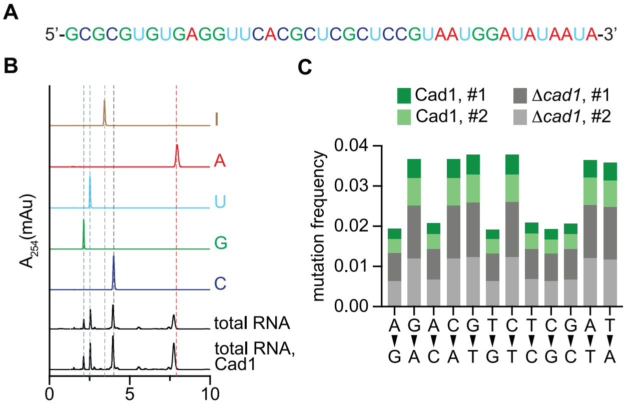

Figure S6. RNA molecules are not affected by Cad1, related to Figure 5

(A) Sequence of the RNA oligonucleotide used as substrate for Cad1.

(B) HPLC analysis of the products of Cad1 activity on the RNA substrate shown in (A) (10 μM RNA) in the presence of cA_6. After treatment, RNA was digested to individual bases using P1 nuclease followed by calf intestinal alkaline phosphatase (CIP). A sample that was not incubated with Cad1 was used as a control. Chromatograms of the different bases are provided as standards.

(C) Analysis of the mutation frequency of staphylococcal transcripts in the presence or absence of Cad1 activation. Mutations were detected by RNA sequencing of two independent experiments, and their number was normalized to the total number of reads.

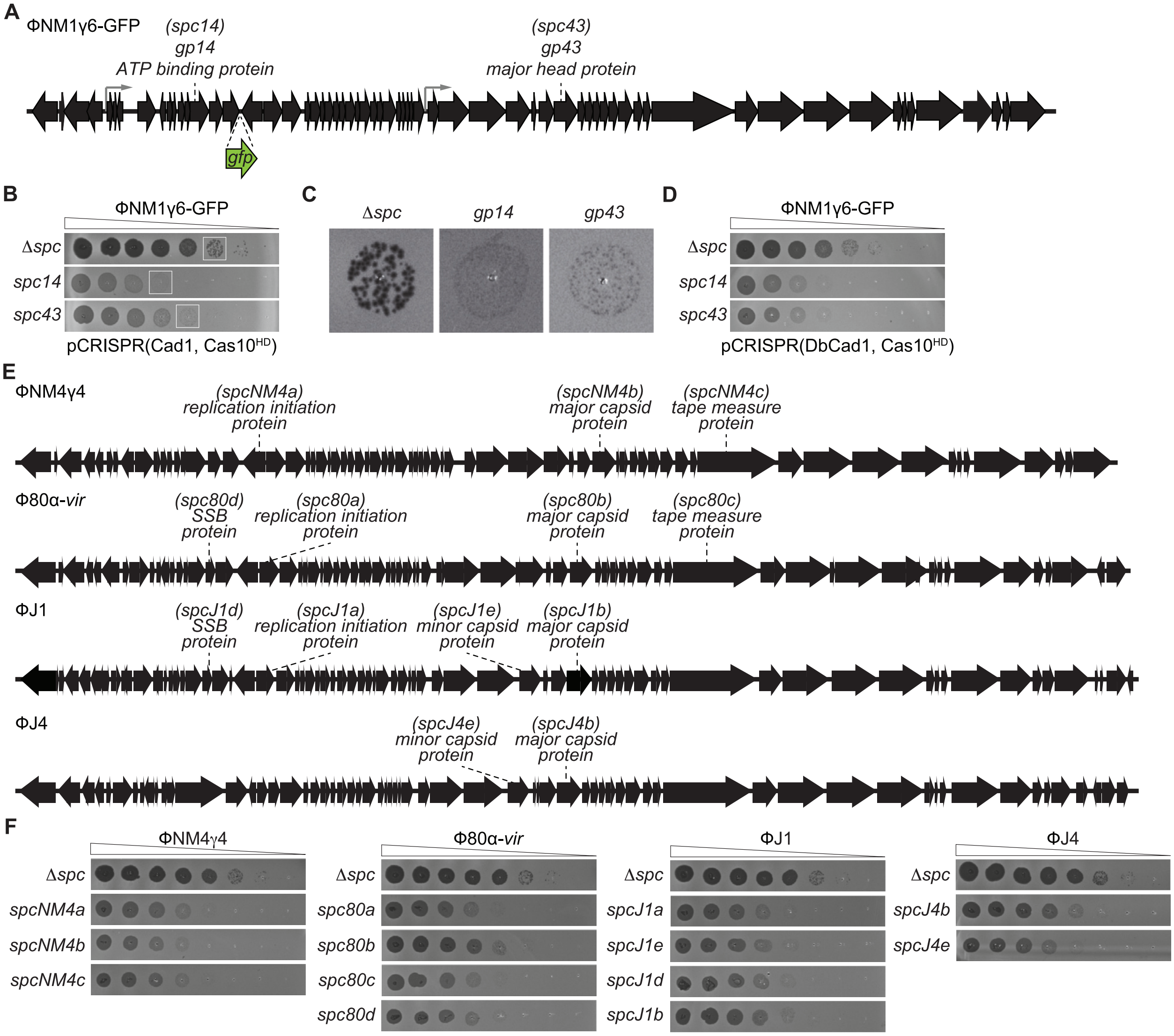

Figure S7. Cad1 immunity against staphylococcal phages, related to Figure 7

(A) Schematic of the genome of the staphylococcal phage ϕNM1γ6-GFP, showing the location of the transcripts targeted by the different spacers of type III-SA CRISPR-Cas system, as well as the insertion site of the *gfp* gene. Left and right gray arrows indicate early and late promoters, respectively.

(B) Plaquing of ΦNM1γ6-GFP on *S. aureus* lawns harboring pCRISPR(Cad1, $Cas10^{HD}$) programmed to target *gp14* or *gp43* with spacers *spc14* or *spc43*, respectively, or a non-targeting control (Δ*spc*). Images are representative of one of three biological triplicates.

(C) Magnification of plaques marked by a white border in (B).

(D) Same as (B) but in the presence of *Desulfomicrobiaceae* bacterium Cad1 (DbCad1). Images are representative of one of three biological triplicates.

(E) Schematic of the genome of the staphylococcal phages ϕNM4γ4, ϕ80α-*vir*, ϕJ1, and ϕJ4, showing the location of the transcripts targeted by the different spacers of type III-SA CRISPR-Cas system.

(F) Plaquing of the phages shown in (E) on *S. aureus* lawns harboring pCRISPR(Cad1, $Cas10^{HD}$) programmed with the indicated spacers, or a non-targeting control (Δ*spc*). Images are representative of one of three biological triplicates.

CellPress
OPEN ACCESS

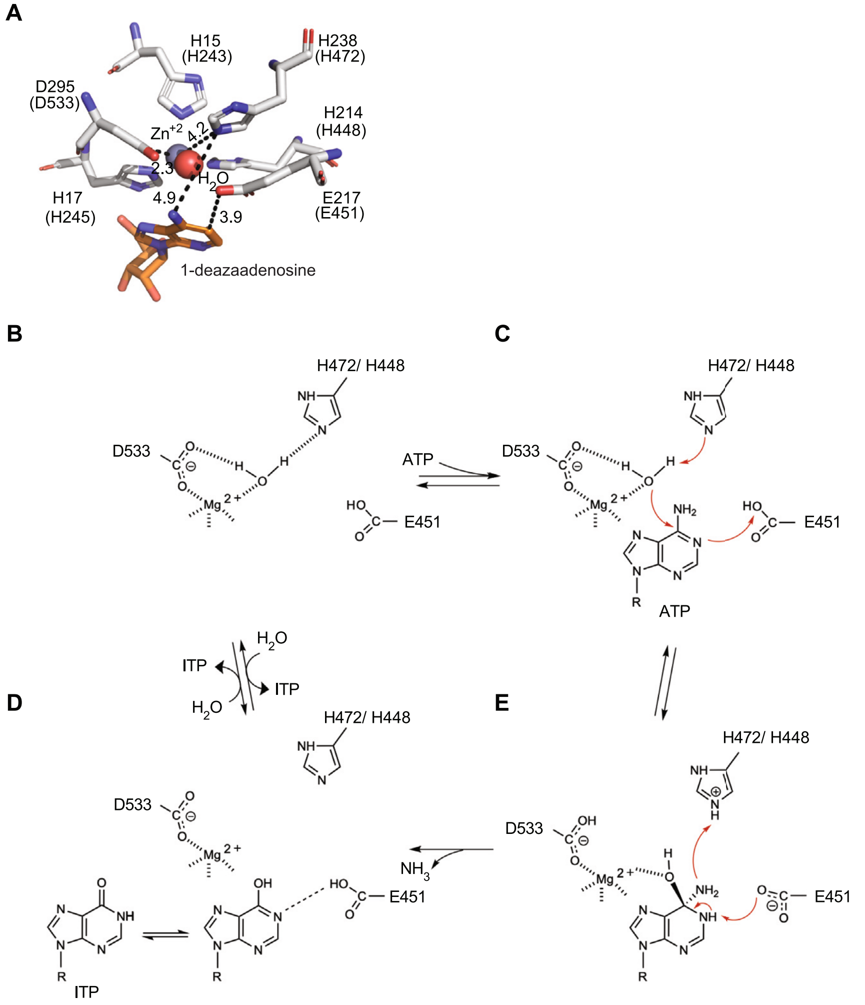

Figure S8. Proposed catalytic mechanism for conversion of ATP to ITP by cOA-activated Cad1, related to Figure 4

(A) Deaminase pocket residues from the crystal structure of murine adenosine deaminase bound to 1-deazaadenosine (PDB-1ADD); distances between different groups are marked by black dotted lines and are measured in Å. Residues in parenthesis correspond to equivalent amino acids in Cad1.

(B) A putative water molecule (not observable in the cryo-EM structure at 3.2 Å resolution) is anchored in place through coordination to a histidine (either H472 or H448), a Mg^{2+} ion, and the carboxylate of D533.

(C) The ATP substrate is positioned such that its adenine N1 is proximal to the carboxylic acid of E451. The histidine abstracts a water proton, thereby activating the OH of water to target the C6 position of the adenine base, which in turn abstracts a proton from E451.

(D) This results in a transient tetrahedral intermediate at the C6 position of the adenine base. The carboxylate of E451 then abstracts the NH at the adenine 1 position, resulting in the release of the NH_2 group from the adenine 6 position, which picks up a proton from the protonated histidine and is released as NH_3.

(E) This results in formation of the enol form of inosine base that is in equilibrium with the keto form.

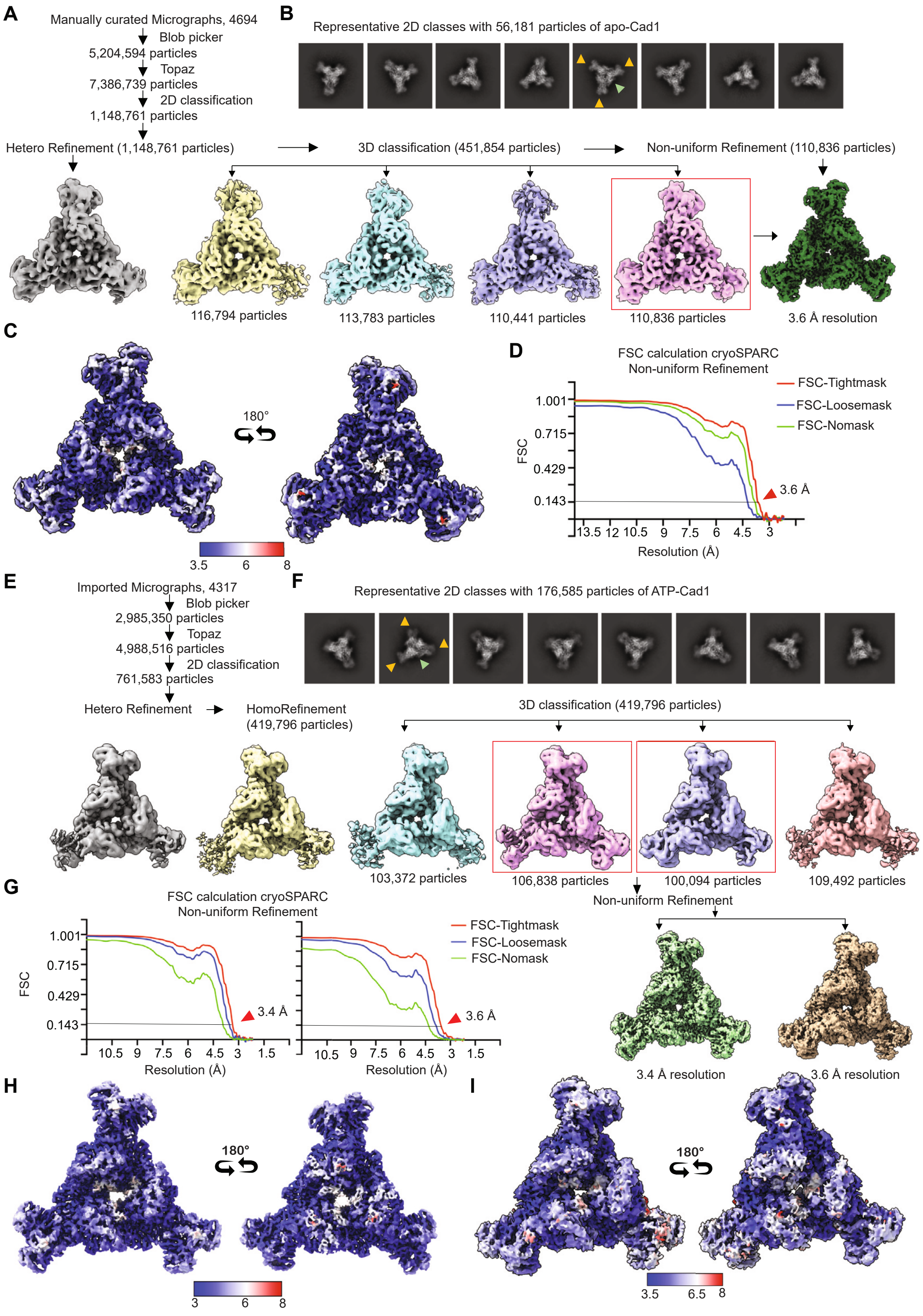

(legend on next page)

CellPress
OPEN ACCESS

Figure S9. Cryo-EM data processing workflow of apo-Cad1 and ATP-bound apo-Cad1 structure determination, related to Figure 3 and STAR Methods

(A) The data processing workflow is indicated, and the number of particles is mentioned for each step. The density map highlighted in red box from the 3D classification job was used for the final round of non-uniform refinement.

(B) Representative 2D classes with 56,181 particles are shown. The CARF dimer head groups are pointed by orange arrows, and the deaminase (ADA) hexameric core is indicated by green arrow.

(C) The local resolution estimated maps are displayed where the scale bar unit is in Å.

(D) The Fourier shell correlation (FSC) curves with the tight, loose, and without mask analyzed by cryoSPARC non-uniform refinement are displayed with the resolution pointed at 0.143 FSC value by red arrow.

(E) The workflow of the ATP bound apo-Cad1 is illustrated with the particle numbers for each step. The two classes marked with the red box in the 3D classification job displayed two different conformations of the protein. Both the classes were refined separately by the final non-uniform refinement jobs.

(F) Representative 2D class averages with 176,585 particles are shown where orange arrows mark the CARF head domains, and the green arrow points out the deaminase (ADA) hexameric core domain.

(G) The Fourier shell correlation (FSC) curves with the tight, loose, and without mask from cryoSPARC non-uniform refinement are displayed for both the refined classes, and the resolution is indicated at FSC value of 0.143 with red arrow.

(H and I) The local resolution estimated maps of class 1 (H) and class 2 (I) are illustrated with front and back views of the maps, and the scale bar is represented in Å.

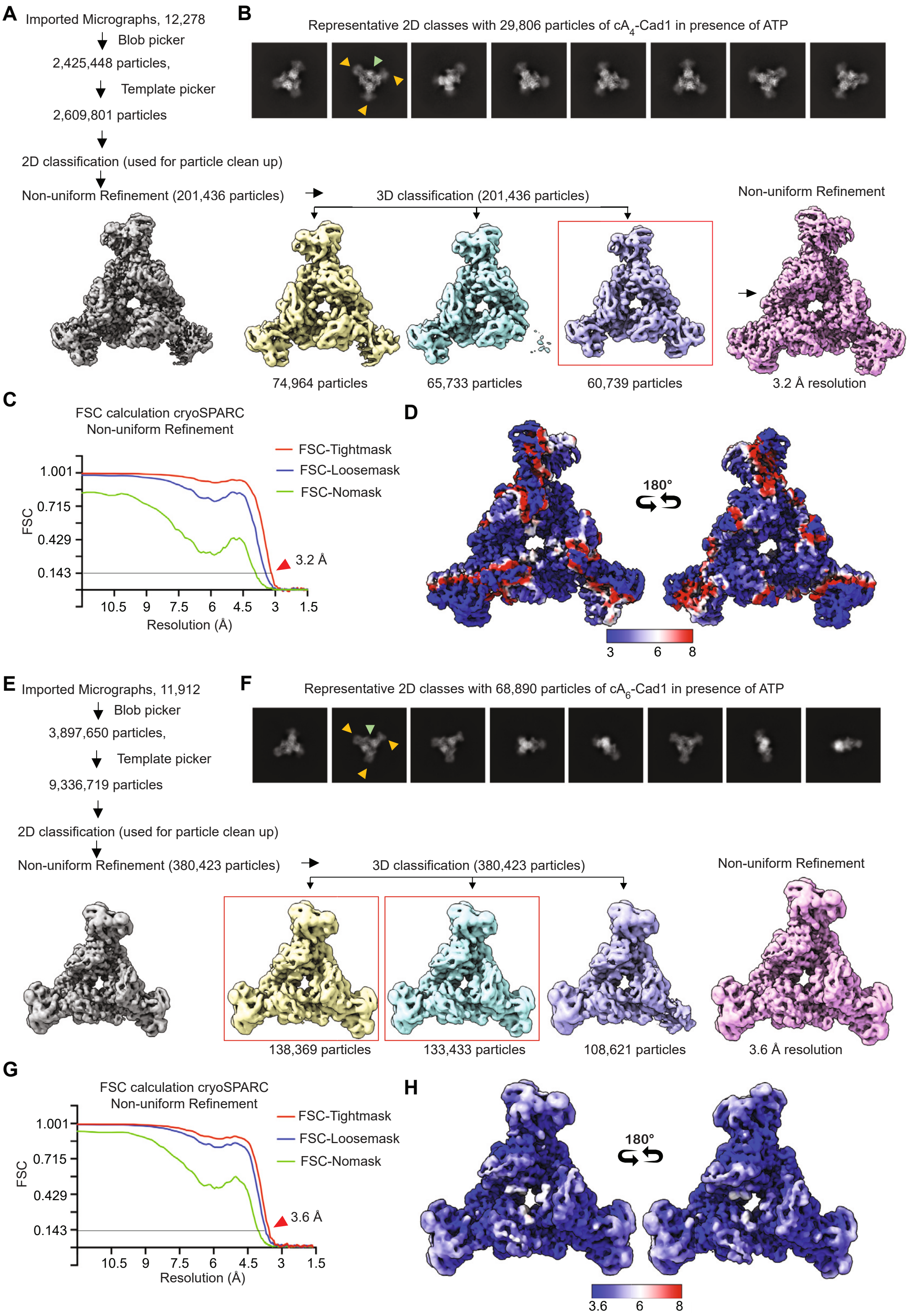

(legend on next page)

Figure S10. Cryo-EM data processing workflow of ATP-bound cA_4-Cad1 and cA_6-Cad1 structure determination, related to Figure 3 and STAR Methods

(A) The data processing flow chart is presented with the particle numbers mentioned for each step. The 3D class, marked with the red box, was selected from the 3D classification job, and the particles were used for the final non-uniform refinement.

(B) Representative 2D class averages are displayed where the CARF domains are pointed with orange arrows, and the deaminase (ADA) hexameric core is pointed with green arrow.

(C) The Fourier shell correlation (FSC) curve with tight mask, loose mask, and without mask displayed in red, blue, and green curves, respectively. The resolution at 0.143 FSC threshold is pointed with a red arrow.

(D) The local resolution estimated EM map is shown in the front and back view. The resolution scale bar is presented in Å.

(E) The presented data-processing flow chart displays the number of particles used for each step. The two 3D classes marked with the red box were selected, and the particles were merged for the final non-uniform refinement job.

(F) The particles with different views are shown using representative 2D classes in which the CARF and the deaminase core domains are marked with orange and green arrows, respectively.

(G) The Fourier shell correlation (FSC) curves with the tight mask (red), loose mask (blue), or without a mask (green) are plotted from the final round of non-uniform refinement job. The red arrow points out the resolution value at 0.143 FSC value.

(H) The local resolution estimation EM map is shown with the front and back views, where the resolution scale bar is represented in Å.
